# Lymph-node-targeted, mKRAS-specific amphiphile vaccine in pancreatic and colorectal cancer: the phase 1 AMPLIFY-201 trial

**DOI:** 10.1038/s41591-023-02760-3

**Published:** 2024-01-09

**Authors:** Shubham Pant, Zev A. Wainberg, Colin D. Weekes, Muhammad Furqan, Pashtoon M. Kasi, Craig E. Devoe, Alexis D. Leal, Vincent Chung, Olca Basturk, Haley VanWyk, Amy M. Tavares, Lochana M. Seenappa, James R. Perry, Thian Kheoh, Lisa K. McNeil, Esther Welkowsky, Peter C. DeMuth, Christopher M. Haqq, Eileen M. O’Reilly

**Affiliations:** 1 The University of Texas MD Anderson Cancer Center, Houston, TX USA; 2 University of California, Los Angeles, Los Angeles, CA USA; 3 Massachusetts General Hospital, Boston, MA USA; 4 University of Iowa, Iowa City, IA USA; 5 Northwell Health, Lake Success, NY USA; 6 University of Colorado School of Medicine, Aurora, CO USA; 7 City of Hope, Duarte, CA USA; 8 Memorial Sloan Kettering Cancer Center, New York, NY USA; 9 Elicio Therapeutics, Boston, MA USA

**Keywords:** Pancreatic cancer, Colon cancer, Adjuvants, Translational immunology

## Abstract

Pancreatic and colorectal cancers are often KRAS mutated and are incurable when tumor DNA or protein persists or recurs after curative intent therapy. Cancer vaccine ELI-002 2P enhances lymph node delivery and immune response using amphiphile (Amph) modification of G12D and G12R mutant KRAS (mKRAS) peptides (Amph-Peptides-2P) together with CpG oligonucleotide adjuvant (Amph-CpG-7909). We treated 25 patients (20 pancreatic and five colorectal) who were positive for minimal residual mKRAS disease (ctDNA and/or serum tumor antigen) after locoregional treatment in a phase 1 study of fixed-dose Amph-Peptides-2P and ascending-dose Amph-CpG-7909; study enrollment is complete with patient follow-up ongoing. Primary endpoints included safety and recommended phase 2 dose (RP2D). The secondary endpoint was tumor biomarker response (longitudinal ctDNA or tumor antigen), with exploratory endpoints including immunogenicity and relapse-free survival (RFS). No dose-limiting toxicities were observed, and the RP2D was 10.0 mg of Amph-CpG-7909. Direct ex vivo mKRAS-specific T cell responses were observed in 21 of 25 patients (84%; 59% both CD4^+^ and CD8^+^); tumor biomarker responses were observed in 21 of 25 patients (84%); biomarker clearance was observed in six of 25 patients (24%; three pancreatic and three colorectal); and the median RFS was 16.33 months. Efficacy correlated with T cell responses above or below the median fold increase over baseline (12.75-fold): median tumor biomarker reduction was −76.0% versus −10.2% (*P* < 0.0014), and the median RFS was not reached versus 4.01 months (hazard ratio = 0.14; *P* = 0.0167). ELI-002 2P was safe and induced considerable T cell responses in patients with immunotherapy-recalcitrant KRAS-mutated tumors. ClinicalTrials.gov identifier: NCT04853017.

## Abstract (web summary)

In a phase 1 trial, a lymph node-targeting mutant KRAS peptide vaccine combined with CpG adjuvant is safe, reduces expression of tumor biomarkers and elicits mutant KRAS-specific T cells in patients with pancreatic cancer and colorectal cancer.

## Main

Colorectal cancer (CRC) and pancreatic ductal adenocarcinoma (PDAC) are the second and third leading causes of cancer death, respectively, with standard locoregional treatment for resectable disease resulting in less than 20% 5-year survival and few effective treatments for relapse^1–4^. PD1 (programmed cell death inhibitor)/PDL1 checkpoint inhibitors alone or in combination with CTLA4 inhibitors are ineffective in PDAC and microsatellite stable (MSS) CRC, consistent with low numbers of antigen-specific tumor-infiltrating lymphocytes (TILs) and infrequent neoantigen mutations^5–8^. However, widely expressed (93% PDAC and 50% CRC) KRAS driver mutations (mKRASs) are attractive immunotherapy targets required for tumor survival with uniform expression throughout disease progression^9,10^. T cells targeting mKRAS are not limited by immune tolerance, and on-target, off-tumor toxicity is unlikely as normal tissues lack expression. Adoptive therapy with HLA C*08:02-restricted KRAS G12D-specific T cells has resulted in responses in patients with PDAC or MSS CRC^11–13^. However, mKRAS-directed T cell therapy is restricted to select mutations and human leukocyte antigen (HLA) alleles, requires complicated logistics, is costly to manufacture and is accompanied by potential cytokine release syndrome and neurotoxicity^11^. In contrast, vaccination offers potential to expand endogenous mKRAS-directed T cells across diverse HLA backgrounds, with simplified manufacturing and off-the-shelf availability.

**Fig. 1:**
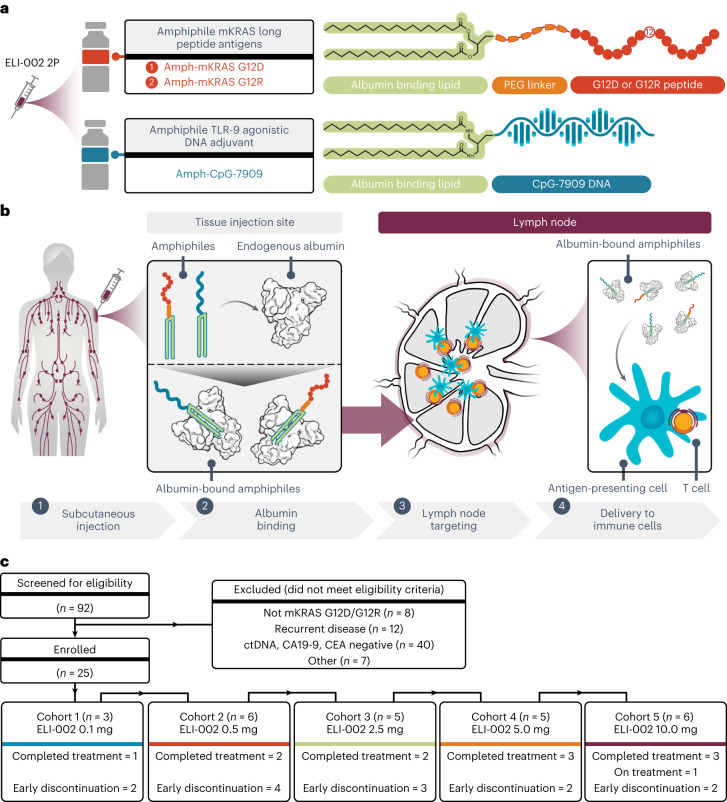
Design of ELI-002 2P, Amph mechanism of action and study participant disposition. **a**, Schematic for ELI-002 2P vaccine components, including Amph-mKRAS G12D and G12R long peptide antigens and Amph-CpG-7909 TLR9 agonist. PEG, polyethylene glycol. **b**, Stepwise schematic for Amph-directed lymph-node-targeted biodistribution mechanism using albumin ‘hitchhiking’: (1) subcutaneous Amph injection, followed by (2) lipid-mediated non-covalent molecular association of Amph vaccines with tissue-resident endogenous albumin, resulting in (3) preferential absorption into lymphatics and accumulation through afferent lymph flow into draining lymph nodes and, finally, (4) uptake of Amph vaccines by lymph-node-resident APCs to induce antigen presentation and coordinated co-stimulation of cognate T cells. **c**, CONSORT diagram. Patients were enrolled into five successive cohorts with progressively increasing doses of Amph-CpG-7909 with a fixed dose of Amph-Peptides 2P. Graphical elements from **a** and **b** were adapted from previous publications under a Creative Commons Attribution 4.0 International License (http://creativecommons.org/licenses/by/4.0/) (refs. ^57,58^).

ELI-002 2P is a three-component lymph-node-targeted vaccine comprising amphiphile (Amph)-modified G12D and G12R mKRAS long peptides as well as Amph-modified Toll-like receptor 9 (TLR9) agonistic CpG-7909 DNA (Fig. 1a and Supplementary Fig. 1). Due to small molecular weight (<20 kDa), conventional peptides and molecular adjuvants in soluble vaccines exhibit poor immunogenicity due to ineffective lymph node biodistribution where resident antigen-presenting cells (APCs) orchestrate immune responses^14^. In contrast, Amph vaccine components (antigens and adjuvants) are modified with diacyl lipids that associate with fatty-acid-binding pockets on endogenous albumin (∼65 kDa) after injection, resulting in molecular ‘hitchhiking’^15^, which enhances lymph node accumulation and efficient delivery into APCs (Fig. 1b)^16,17^. Preclinically, Amph vaccination delivered vaccine components to and activated resident APCs, robustly reprogramming the immune microenvironment to develop high-magnitude, functional T cell responses^15,18,19^. Amph vaccine therapy enhanced efficacy with increased T cell expansion and tumor infiltration, leading to long-term eradication of solid tumors alongside decreased systemic toxicity^20,21^.

**Extended Data Fig. 1:**
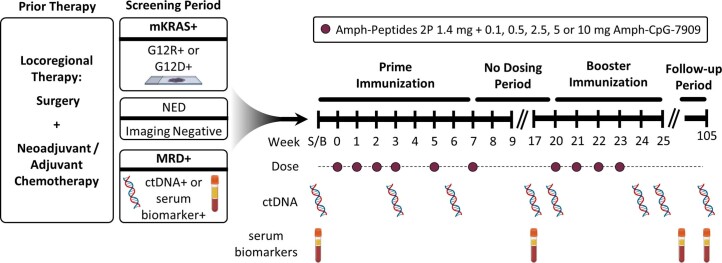
Study schema. Treatment with ELI-002 2P was conducted in a priming immunization period (B: baseline – week 7) and subsequent boosting immunization period (week 20-23) for CRC or PDAC patients exhibiting tumor expression of mKRAS G12D or G12R following completion of locoregional therapy, radiological confirmation of no evidence of disease, and detection of either ctDNA or serum tumor biomarker indicating positivity for minimal residual disease (MRD).

KRAS-mutated pancreatic tumors exhibit total loss of class I HLA expression in 43% of metastases but in only 6% of primary tumors^22^. We hypothesized that administration of ELI-002 2P after tumor resection in the setting of minimal residual disease (MRD) would (1) induce expansion of functional tumor-directed mKRAS-specific T cells, (2) increase the potential for anti-tumor activity by avoiding HLA loss in late-stage disease and (3) allow T cell action to support destruction of micro-metastatic disease, resulting in reduced tumor biomarkers and delayed radiographic recurrence (Extended Data Fig. 1). Here we report the safety, efficacy, immunogenicity and recommended phase 2 dose (RP2D) of ELI-002 2P in a phase 1 multi-cohort study, AMPLIFY-201, in patients who had received definitive locoregional therapy but were positive for MRD with high risk for radiographic relapse due to the presence of circulating tumor DNA (ctDNA) or serum tumor antigen biomarkers.

## Results

### Patients

Twenty-five patients were enrolled and initiated dosing between 4 October 2021 and 10 April 2023 with ELI-002 2P (Table 1), comprising a fixed dose of Amph-Peptides 2P (0.7 mg of G12D and G12R antigens each), with escalating Amph-CpG-7909 doses of 0.1, 0.5, 2.5, 5.0 and 10.0 mg in cohorts 1, 2, 3, 4 and 5, respectively, using a modified 3 + 3 design. Enrollment required (1) presence of a tumor mKRAS mutation (G12D or G12R) determined by whole-exome sequencing, (2) imaging studies negative for overt disease and (3) MRD state as adjudicated by detection of ctDNA positivity and/or elevated serum tumor antigen, carbohydrate antigen (CA) 19-9 or carcinoembryonic antigen (CEA) (Extended Data Fig. 1 and Extended Data Table 1). A tumor-informed ctDNA assay was used to follow 21 of 25 patients, and a serum-based ctDNA assay was used in four of 25 patients when a tumor-informed assay was not feasible. Per the protocol design, up to three additional patients (total of up to six per cohort) were permitted into each dose level even in the absence of a dose-limiting toxicity (DLT) for any cohort previously deemed safe and well tolerated by the safety review committee overseeing the study. As of the date of data cutoff, 6 September 2023, all 25 enrolled patients were included in the analyses of safety, immunogenicity and preliminary anti-tumor activity through evaluation of ctDNA and serum tumor antigens and relapse-free survival (RFS) (Fig. 1c). Thirteen patients discontinued treatment early due to disease progression (two, four, three, two and two in cohorts 1, 2, 3, 4 and 5, respectively), and no patient discontinued study treatment due to toxicity. Concomitant anti-tumor therapy was prohibited during study treatment, and the details of subsequent therapy were collected for patients entering the 2-year long-term follow-up period after completing ELI-002 treatment or early discontinuation. Baseline characteristics (Table 1) included median age 61.0 years (range, 37–77 years), and most patients were female (60%) and White (84%). Twenty patients (80%) had PDAC, and five patients (20%) had CRC. Prior surgical pathology demonstrated that 68% of patients had stage III or resected oligometastatic (≤3 lesions in one organ) stage IV disease, with only six (24%) being node negative (TNM; Extended Data Table 1). All patients had received prior chemotherapy and anti-cancer surgery, and 28% had received prior radiation therapy (Extended Data Table 1).

**Table 1:** Demographics and baseline characteristics of study participants

	Cohort 1 (0.1 mg) *n* = 3	Cohort 2 (0.5 mg) *n* = 6	Cohort 3 (2.5 mg) *n* = 5	Cohort 4 (5.0 mg) *n* = 5	Cohort 5 (10.0 mg) *n* = 6	Overall *n* = 25
Age (years)						
Median	50.0	54.5	67.0	67.0	59.0	61.0
Range	47–61	37–69	63–77	47–75	48–69	37–77
Female sex, *n* (%)	2 (66.7)	5 (83.3)	4 (80.0)	3 (60.0)	1 (16.7)	15 (60.0)
BMI (kg/m^2^)						
Median	25.2	22.1	22.3	23.3	26.2	23.4
Range	20.5–32.2	16.5–38.0	19.9–24.6	16.0–31.1	19.1–33.3	16.0–38.0
Race, *n* (%)						
Asian	0	1 (16.7)	0	0	1 (16.7)	2 (8.0)
White	2 (66.7)	5 (83.3)	4 (80.0)	5 (100)	5 (83.3)	21 (84.0)
Not reported	1 (33.3)	0	1 (20)	0	0	2 (8.0)
ECOG PS, *n* (%)						
0	3 (100)	4 (66.7)	4 (80.0)	2 (40.0)	5 (83.3)	18 (72.0)
1	0	2 (33.3)	1 (20.0)	3 (60.0)	1 (16.7)	7 (28.0)
Initial diagnosis, *n* (%)						
PDAC	1 (33.3)	4 (66.7)	5 (100)	4 (80.0)	6 (100)	20 (80.0)
CRC	2 (66.7)	2 (33.3)	0	1 (20.0)	0	5 (20.0)
Disease stage at screening						
Stage I, II	1 (33.3)	2 (33.3)	3 (60.0)	1 (20.0)	1 (16.7)	8 (32.0)
Stage III, IV^a^	2 (66.7)	4 (66.7)	2 (40.0)	4 (80.0)	5 (83.3)	17 (68.0)
Prior anti-cancer therapy						
Systemic therapy	3 (100)	6 (100)	5 (100)	5 (100)	6 (100)	25 (100)
Surgery/procedure	3 (100)	6 (100)	5 (100)	5 (100)	6 (100)	25 (100)
Radiation therapy	2 (66.7)	1 (16.7)	1 (20.0)	2 (40.0)	1 (16.7)	7 (28.0)
Tumor KRAS mutation						
G12 amino acid	DDD	DDDDDD	DRDDD	DDRDD	RRDDRD	

BMI, body mass index.

^a^Resected oligometastatic (<3 lesions) with NED on imaging was permitted.

### Safety

The primary endpoint was to evaluate the safety of ELI-002 2P and to identify the maximum tolerated dose (MTD) and the RP2D. Forty-eight percent of patients (12/25) experienced an adverse event related to ELI-002 2P (Table 2), all grade 1 or grade 2; there were no grade ≥3–related events, no cytokine release syndrome and no DLTs, and no MTD was established. Related treatment-emergent adverse events reported for ≥3 patients were fatigue (*n* = 6, 24%, five grade 1 and one grade 2), injection site reaction (*n* = 4, 16%, all grade 1) and myalgia (*n* = 3, 12%, all grade 1). One serious adverse event was reported: a grade 3 abdominal wall hematoma that resolved 8 d after onset was considered related to a biopsy performed per protocol to confirm progression and unrelated to ELI-002. No event led to discontinuation of treatment or death. Patient diaries were used to assess injection site reactogenicity and systemic reactogenicity symptoms (Extended Data Table 2); 92% (23/25) recorded mild symptoms, 56% (14/25) recorded moderate symptoms and 12% (3/25) recorded severe symptoms. The RP2D was determined to be 10 mg of Amph-CpG-7909, fulfilling the primary study endpoint, as this dose was safe and well tolerated and was associated with consistent biomarker reductions and T cell responses.

**Table 2:** Adverse events and summary for study participants

	Cohort 1 (0.1 mg) *n* = 3	Cohort 2 (0.5 mg) *n* = 6	Cohort 3 (2.5 mg) *n* = 5	Cohort 4 (5.0 mg) *n* = 5	Cohort 5 (10.0 mg) *n* = 6	Overall *n* = 25
**Adverse event term**^a^						
Patients with any related TEAE, *n* (%)	1 (33.3)	3 (50.0)	2 (40.0)	2 (40.0)	4 (66.7)	12 (48.0)
Fatigue	0	2 (33.3)	2 (40.0)	1 (20.0)	1 (16.7)	6 (24.0)
Injection site reaction*	1 (33.3)	1 (16.7)	0	2 (40.0)	0	4 (16.0)
Myalgia	0	0	0	1 (20.0)	2 (33.3)	3 (12.0)
Anemia	1 (33.3)	0	1 (20.0)	0	0	2 (8.0)
Headache	1 (33.3)	1 (16.7)	0	0	0	2 (8.0)
Hot flush	0	1 (16.7)	0	0	1 (16.7)	2 (8.0)
Nasal congestion	0	1 (16.7)	0	1 (20.0)	0	2 (8.0)
Nausea	1 (33.3)	0	0	1 (20.0)	0	2 (8.0)

**Patient summary**^b^						
KRAS mutation	DDD	DDDDDD	DRDDD	DDRDD	RRDDRD	
DLT	0	0	0	0	0	0
Biomarker reduction/clearance	2 (67)	5 (83)	4 (80)	4 (80)	6 (100)	21 (84)
T cell response	2 (67)	4 (67)	4 (80)	5 (100)	6 (100)	21 (84)

^a^TEAE, treatment-emergent adverse events with incidence ≥5%; data cutoff, 6 September 2023; preferred terms per the Medical Dictionary for Regulatory Activities, version 25.0

* Injection site reaction includes injection site erythema, injection site induration, injection site swelling, contusion and pruritis.

^b^ Patient summary.

### Tumor biomarker response

**Fig. 2:**
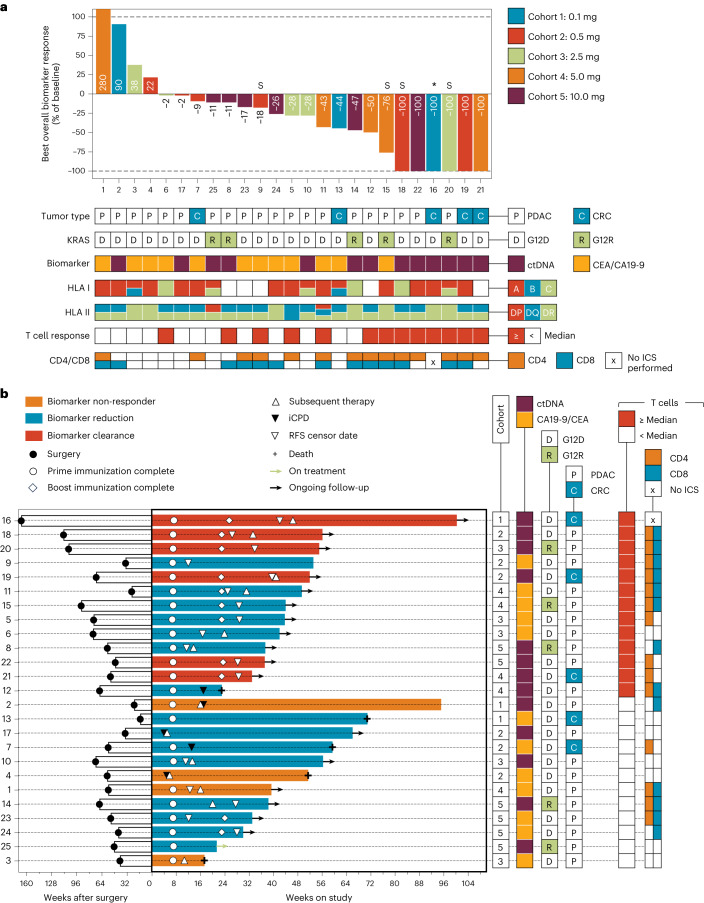
ELI-002 2P treatment results in tumor biomarker reduction and clearance. **a**, Best overall tumor biomarker response for study participants reported as percentage of the baseline value. Responses are annotated for patient number, dose cohort, participant tumor type, KRAS mutation, tumor biomarker type and presence or absence of previously reported mKRAS-responsive HLA class I and II. * Patient 16 underwent excisional biopsy to assess iRECIST, and T cell infiltrate was observed; a contribution to ctDNA clearance could not be ruled out. ‘S’ indicates that the patient underwent splenectomy. **b**, Swimmer plot supervised by T cell response (maximum fold change from baseline; top: ≥median; bottom: <median) showing time from surgery to start of ELI-002 2P (left) and time from start of ELI-002 2P to endpoint (right; data cutoff or death), *n* = 25. Bar color indicates best overall tumor biomarker response for each patient. Symbols annotate the timing of completion of prime and boost immunizations, the start of subsequent therapy, iCPD, the most recent date of censor for RFS (the date of the most recent radiographic scan before the date of subsequent therapy, the data cutoff or the date of death) and the date of death where applicable. Arrows indicate ongoing treatment or ongoing follow-up. iCPD, immune confirmed progressive disease.

Tumor biomarker response, the secondary endpoint, was assessed as any decrease from baseline in ctDNA and/or serum tumor antigen levels (CA19-9 and CEA) irrespective of subsequent biomarker values. In the biomarker-evaluable study population, 84% of patients (21/25; Fig. 2a and Extended Data Table 3) had a decline from baseline: 44% (11/25) had a ≥30% drop; 32% (8/25) had a ≥50% drop; and 24% (6/25; *n* = 3 pancreatic and *n* = 3 colorectal) had complete biomarker clearance (0 mean tumor molecules per milliliter (MTM/ml) for ctDNA, and five of six clearances were confirmed by subsequent undetectable ctDNA values (Extended Data Table 3)). Serum CEA or CA19-9 declines contributed to tumor biomarker response but not clearance because results remained above the limit of detection for these assays.

Similar proportions of patients exhibited reductions from baseline assessed using CEA/CA19-9 (75%, 9/12) or ctDNA (92%, 12/13). Likewise, patients with PDAC (80%, 16/20) and CRC (100%, 5/5) exhibited biomarker declines. All patients with G12R mutations (100%, 5/5) and 80% (16/20) of those with G12D mutations demonstrated a biomarker decline. Responses occurred in four G12D patients (all four assessed using ctDNA) without any class I HLA alleles previously reported to restrict G12D T cell responses (bioRxiv 2020.06.15.149021)^11,23,24^. Likewise, all five patients with G12R-mutated tumors had biomarker responses despite lacking HLA B*07:02 previously reported to restrict G12R T cell responses. Similar proportions of males (80%, 8/10) and females (87%, 13/15) exhibited tumor biomarker responses. All patients had at least one class II HLA allele previously reported to restrict CD4^+^ T cell responses to mKRAS^11,23,24^. Responses were observed across all durations (median 5.7 months; range, 2.6–31.9 months) from prior surgery or chemotherapy (whichever was last). Notably, all who had undergone prior splenectomy during the surgical management of their primary pancreatic tumor (100%, 4/4; ‘S’ annotation in Fig. 2a) responded, with 50% (2/4) exhibiting tumor biomarker clearance.

**Extended Data Fig. 2:**
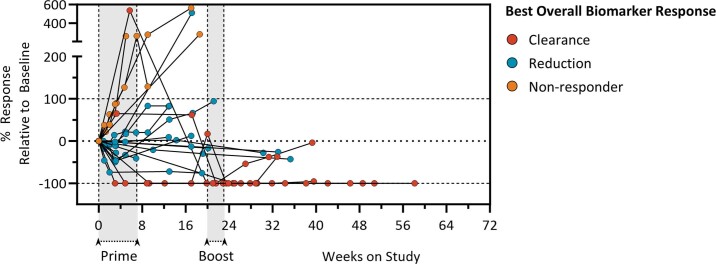
ELI-002 2P treatment results in tumor biomarker reduction and clearance. Longitudinal changes in biomarker levels as a percentage of baseline values for each evaluable patient, *n* = 25. Responses are annotated based on the best overall biomarker response observed for each patient during the course of study.

Extended Data Fig. 2 illustrates the exploratory kinetics of biomarker changes from baseline in a spider plot. Non-responders generally had progressive biomarker increases; in six of 15 (40%) patients with biomarker reduction, response was transient and followed by progression, whereas responses were ongoing in the other nine of 15 (60%); and another two patients had initial rising biomarker values followed by reduction to undetectable levels, indicating that a minority of patients may exhibit initial tumor biomarker increases before progressive reductions. Patient 16 with the longest follow-up (CRC) had three prior systemic adjuvant chemotherapies plus radiation therapy but was ctDNA positive throughout prior treatment and in screening but achieved clearance of ctDNA after treatment with ELI-002 2P and remained no evidence of disease (NED) on imaging throughout follow-up (Extended Data Fig. 2).

A swimmer’s plot for the biomarker-evaluable population is illustrated as Fig. 2b. The median duration of study treatment was 20.1 weeks, with 23 of 25 patients completing the prime series, 11 of 25 patients proceeding to the booster phase and 11 of 25 patients completing all specified protocol therapy; one patient was continuing treatment at the database cutoff.

### Immunogenicity and clinical outcomes

**Fig. 3:**
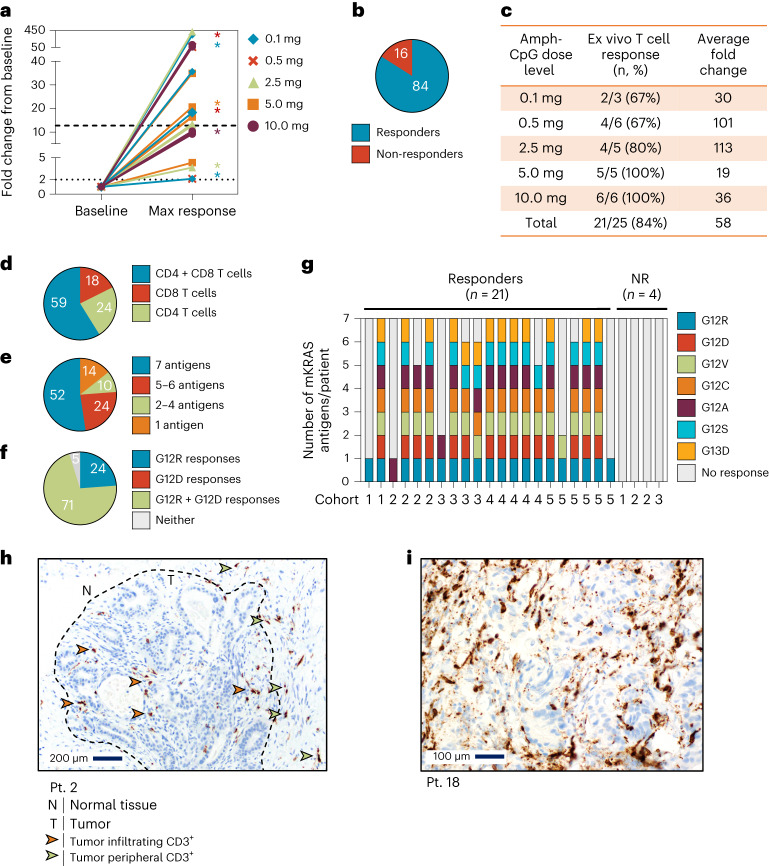
Robust direct ex vivo mKRAS-specific T cell responses induced by ELI-002 2P in a majority of patients. Patients were immunized with 1.4 mg of Amph-Peptides 2P admixed with 0.1, 0.5, 2.5, 5 or 10 mg of Amph-CpG-7909. PBMCs were collected for T cell response assessment at baseline and post-immunization timepoints. **a**, Shown is the fold change from baseline to maximum response in the ex vivo FluoroSpot assay or ICS assay for T cell responders (*n* = 21/25 patients). T cell responders are defined as patients having a ≥2-fold increase from baseline at any post-vaccination timepoint (dotted line) and >50 SFCs/1 × 10^6^ PBMCs for FluoroSpot assay or >0.1% cytokine-positive for ICS assay. The median fold change is shown at 12.75, indicated by a dashed line. * Patients without baseline-detectable T cell responses (below the response threshold) had on-treatment fold change of 348×, 58.3×, 20.7×, 18.3×, 9.7×, 3.7× and 2.1×. **b**, Pie chart shows the percentage of T cell responders and non-responders to ex vivo FluoroSpot and ICS. **c**, Table shows the percentage of T cell responders to ex vivo FluoroSpot and ICS and average fold change of T cell responders per dose cohort. **d**, Pie chart depicts the percentage of T cell responders that induce CD4^+^, CD8^+^ or both CD4^+^ and CD8^+^ cytokine-positive cells in ICS assay. **e**, Pie chart indicates the percentage of T cell responders that induce T cell responses to 1, 2–4, 5–6 or all 7 assessed mKRAS antigens. **f**, Pie chart shows the percentage of T cell responders that induce T cell responses to immunizing antigens G12D and/or G12R. **g**, Bar graph indicates the number of mKRAS antigens that induced T cell responses after vaccination for each patient, *n* = 25. **h**, CD3 immunohistochemical analysis of hepatic tumor biopsy tissue section collected from patient 2 after observation of a contrast avid lesion by computed tomography (CT) 33 d after the start of treatment. Twenty-four CD3^+^ T cells per high-powered field (×400) were quantitated by the study pathologist in the region of highest labeling. **i**, CD3 immunohistochemical analysis of hepatic tumor biopsy tissue section collected from patient 18 after observation of a contrast avid lesion by CT. Seventy-six CD3^+^ T cells per high-powered field (×400) were quantitated by the study pathologist in the region of highest labeling. Pt., patient.

**Extended Data Fig. 3:**
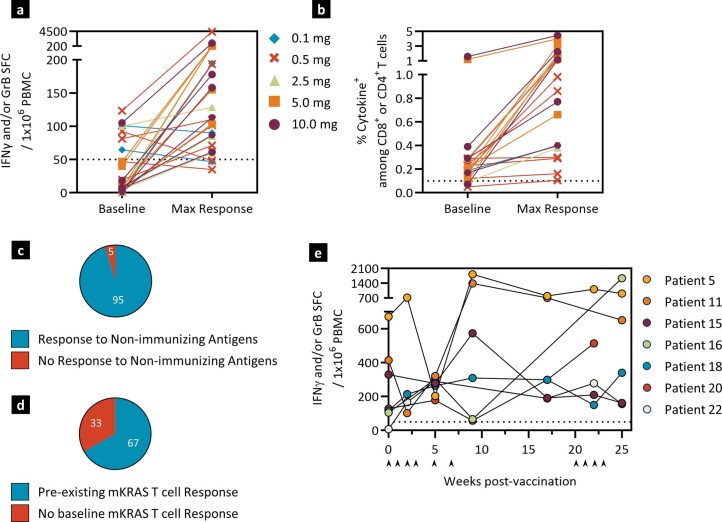
Robust direct ex vivo mKRAS-specific T cell responses induced by ELI-002 2P in a majority of patients. Patients were immunized with 1.4 mg Amph-Peptides 2P admixed with 0.1, 0.5, 2.5, 5 or 10 mg of Amph-CpG-7909. PBMCs were collected for T cell response assessment at baseline and post-immunization timepoints. **a**, PBMCs were stimulated with OLPs to all 7 mKRAS antigens in the IFNγ/GrB Fluorospot assay for 44 hours. Shown are background-subtracted IFNγ and/or GrB SFCs per 1 × 10^6^ PBMCs for the maximum response for each patient. T cell responders are defined as a patients having ≥2-fold increase from baseline at any post-vaccination timepoint and >50 SFC/1×10^6^ PBMCs (dotted line). **b**, PBMCs were stimulated with all 7 mKRAS OLPs prior to analysis for intracellular cytokines, IFNγ, TNFa and IL-2 by flow cytometry. Shown are frequencies of the maximum cytokine producing CD4+ or CD8+ T cells for each patient. T cell responders are defined as a patients having ≥2-fold increase from baseline at any post-vaccination timepoint and > 0.1% cytokine+ (dotted line). **c**, Pie chart shows the percentage of T cell responders that induce T cell responses to non-immunizing antigens (G12V, G12C, G12A, G12S, G13D). **d**, Pie chart depicts the percentage of T cell responders exhibiting pre-existing mKRAS-specific T cells seen at baseline before ELI-002 2P vaccination, *n* = 21. **e**, PBMCs were stimulated with OLPs to all 7 mKRAS antigens in the IFNγ/GrB Fluorospot assay for 44 hours. Shown are background-subtracted IFNγ and/or GrB SFCs per 1 × 10^6^ PBMCs for the 7 patients with post-booster vaccination timepoints. The arrows on the graph indicate the prime and boost vaccinations; dotted line indicates 50 SFC/1 x 10^6^ PBMCs.

**Extended Data Fig. 4:**
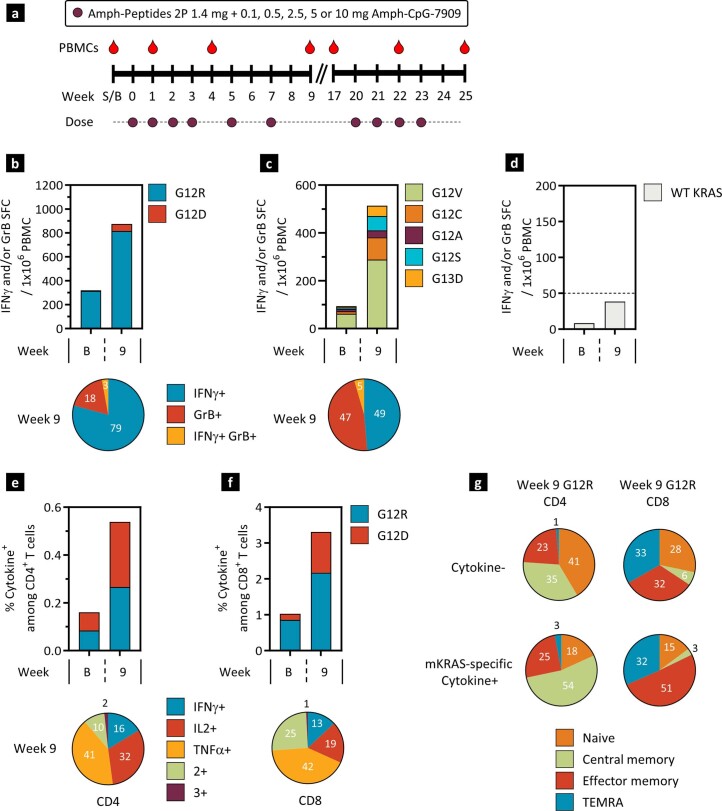
ELI-002 2P immunization induces robust T cell responses to mKRAS antigens. Patient 11 was immunized with 1.4 mg Amph-Peptides 2P admixed with 5 mg of Amph-CpG-7909. PBMCs were collected for ex vivo T cell response assessments at baseline (B) and week 9. **a**, Schema showing dosing and experimental schedule. PBMCs were stimulated with OLPs to **b**, G12D or G12R, **c**, G12V, G12C, G12A, G12S or G13D or **d**, WT KRAS in the same IFNγ/GrB Fluorospot assay for 44 hours. Shown are background-subtracted IFNγ and/or GrB SFCs per 1 × 10^6^ PBMCs; dotted line indicates 50 SFC/1 x 10^6^ PBMCs. The pie charts in **b, c** show the percentage of mKRAS OLP-stimulated PBMCs at week 9 secreting IFNγ, GrB or both IFNγ and GrB. **e, f**, PBMCs were stimulated with G12R or G12D OLPs prior to analysis for intracellular cytokines, IFNγ, TNFa and IL-2 by flow cytometry. Shown are frequencies of total IL2, TNFα, IFNγ polyfunctional cytokine producing **e**, CD4+ and **f**, CD8+ T cells. The pie charts in **e, f** show the percentage of G12R and G12D stimulated PBMCs producing IFNγ, IL2, TNFα, producing 2 cytokines (2+) or producing 3 cytokines (3+) at week 9. **g**, The pie charts depict the percentage of G12R-stimulated memory CD4+ and CD8+ T cells in cytokine negative and cytokine positive cells at week 9 (Naïve: CCR7+ CD45RA+, Central memory: CCR7 + CD45RA-, Effector memory: CCR7- CD45RA-, Terminal effector memory: CCR7- CD45RA+).

**Extended Data Fig. 5:**
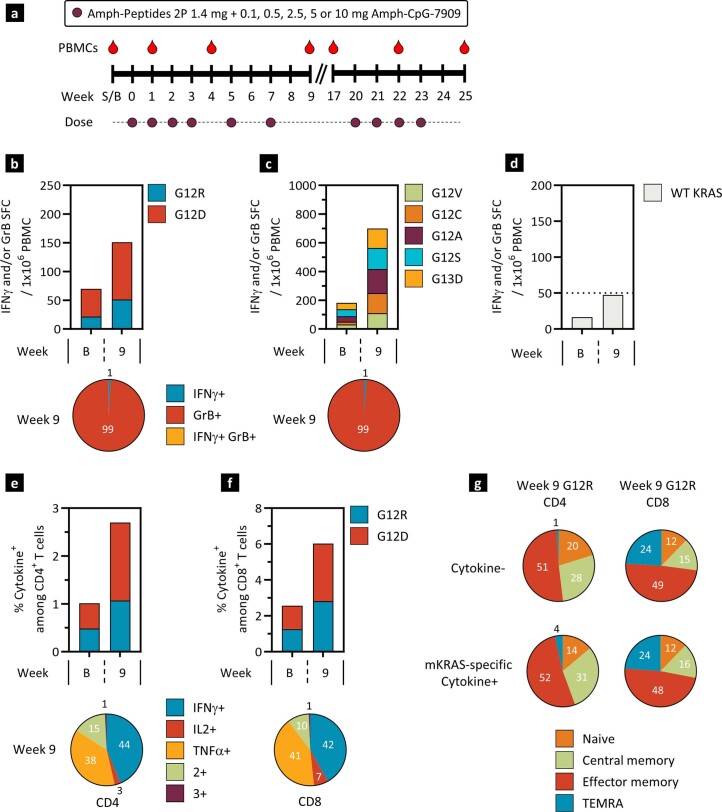
ELI-002 immunization induces robust T cell responses to mKRAS antigens. Patient 23 was immunized with 1.4 mg Amph-Peptides 2P admixed with 10 mg of Amph-CpG-7909. PBMCs were collected for ex vivo T cell response assessments at baseline (B) and Week 9. **a**, Schema showing dosing and experimental schedule. PBMCs were stimulated with OLPs to **b**, G12D or G12R, **c**, G12V, G12C, G12A, G12S or G13D or **d**, WT KRAS in the same IFNγ/GrB Fluorospot assay for 44 hours; dotted line indicates 50 SFC/1 x 10^6^ PBMCs. Shown are background-subtracted IFNγ and/or GrB SFCs per 1 × 10^6^ PBMCs. The pie charts in **b, c** show the percentage of mKRAS OLP-stimulated PBMCs at week 9 secreting IFNγ, GrB or both IFNγ and GrB. **e, f**, PBMCs were stimulated with G12R or G12D OLPs prior to analysis for intracellular cytokines, IFNγ, TNFa and IL-2 by flow cytometry. Shown are frequencies of total IL2, TNFα, IFNγ polyfunctional cytokine producing **e**, CD4+ and **f**, CD8+ T cells. The pie charts in **e, f** show the percentage of G12R and G12D stimulated PBMCs producing IFNγ, IL2, TNFα, producing 2 cytokines (2+) or producing 3 cytokines (3+) at week 9. **g**, The pie charts depict the percentage of G12R-stimulated memory CD4+ and CD8+ T cells in cytokine negative and cytokine positive cells at week 9 (Naïve: CCR7 + CD45RA+, Central memory: CCR7 + CD45RA-, Effector memory: CCR7- CD45RA-, Terminal effector memory: CCR7- CD45RA+).

The exploratory immunogenicity of ELI-002 2P was assessed directly ex vivo in the peripheral blood of patients throughout the study (Fig. 3a and Extended Data Fig. 3). Peripheral blood mononuclear cells (PBMCs) were stimulated individually for all mKRAS antigens and wild-type (WT) antigen and directly measured for T cell responses. Example T cell responses for patient 11 (tumor mKRAS G12D) are shown in Extended Data Fig. 4. Elevated T cell immunity to the vaccine epitopes G12R and G12D was observed after the prime immunization series (Extended Data Fig. 4a) at week 9 by ex vivo IFNγ/Granzyme B (GrB) FluoroSpot, with 2.6-fold and 12-fold increases over baseline, respectively (Extended Data Fig. 4b). Seventy-nine percent of T cells secreted IFNγ, and 18% secreted cytolytic marker GrB. Consistent with previous reports^25–28^, baseline antigen-specific T cell responses to G12R and G12D were also observed; however, ELI-002 2P vaccination greatly expanded the frequency of these pre-existing T cells to over 800 spot-forming cells (SFCs) per million. T cell responses specific to other mKRAS antigens were also evaluated, and positive immune responses (defined as >2-fold over baseline and at least 50 SFCs) were induced to G12V, G12C and G12S but not WT KRAS (Extended Data Fig. 4c,d). Polyfunctional CD4^+^ and CD8^+^ T cell responses were observed to G12R and G12D after vaccination in an ex vivo intracellular cytokine staining (ICS) assay for IFNγ, TNFα and IL2 (Extended Data Fig. 4e,f). Twelve percent of CD4^+^ T cells and 28% of CD8^+^ T cells were polyfunctional, simultaneously producing two or three cytokines. Furthermore, most cytokine-positive CD4^+^ and CD8^+^ T cells were central and effector memory T cells (Extended Data Fig. 4g). Similar results for patient 23 are included in Extended Data Fig. 5. Together, these results indicate the development of a robust, mKRAS-specific, polyfunctional CD4^+^ and CD8^+^ T cell response accompanied by formation of a memory compartment after immunization with ELI-002 2P that was greatly amplified from baseline.

The overall immunogenicity of ELI-002 2P was assessed in 25 patients from all five dose-level cohorts of Amph-CpG-7909, with direct ex vivo T cell responses observed in 84% of patients (21/25; Fig. 3a,b and Extended Data Fig. 3). Fold change increases ranged from twofold to 423-fold, and the average increase was 58-fold. At the two highest dose levels, 5 mg and 10 mg of Amph-CpG-7909, all patients (11/11, 100%) demonstrated elevated mKRAS-specific T cell responses after ELI-002 2P vaccination (Fig. 3c). All patients with prior splenectomy (4/4, 100%) exhibited T cell responses as did all evaluable non-White patients (2/2, 100%). In the ICS assay, 59% of patients showed a mKRAS-specific response including both CD4^+^ and CD8^+^ T cells (Fig. 3d). Ex vivo FluoroSpot and ICS assays were used to assess the breadth of mKRAS-specific T cell responses (Fig. 3e–g). Eighty-six percent of immune responders (18/21) had T cell responses to ≥2 mKRAS antigens (Fig. 3e,g). Furthermore, 52% of immune responders (11/21) induced T cell responses to all seven mKRAS antigens tested (Fig. 3e,g). Notably, 95% of patients developed responses to at least one mKRAS antigen distinct from the immunizing G12R and G12D epitopes (Extended Data Fig. 3), and 95% of immune responders induced T cell responses to the vaccine antigens, G12R and/or G12D (Fig. 3g). Among the 25 study patients immunized with two antigens (G12D and G12R), ex vivo responses were induced to 30 of 50 of the included vaccine epitopes (60%). Among the 21 immune responders, baseline responses indicated that 67% (14/21) of immune responders had detectable pre-existing mKRAS-specific T cells before ELI-002 2P vaccination (Extended Data Fig. 3); however, immune response was also observed in 100% (7/7) of patients without detectable pre-existing mKRAS-specific T cells at baseline, indicating the potential for de novo priming as well as expansion of existing T cell responses by ELI-002 2P (Fig. 3a). Longitudinal assessment of T cell responses was evaluable in seven patients entering the booster immunization period at the time of database cutoff; 86% (6/7) of patients exhibited either maintenance of elevated T cell response relative to baseline levels or increased response after boost (Extended Data Fig. 3e).

In six patients, tumor biopsies performed at the time of radiographic progression provided the potential to observe the presence of infiltrating T cell responses after ELI-002 2P treatment. Although comparison of T cell infiltration in matched pre-treatment metastatic samples was not possible because eligibility required no baseline radiographic disease, progression on ELI-002 did not appear to be mediated by poor T cell tumor infiltration (biopsies showed 24, 25, 32, 43, 58 and 76 CD3^+^ T cells per high-powered field; Extended Data Table 2). For example, in patient 2 (PDAC) treated on cohort 1 (0.1-mg Amph-CpG dose level), biopsy was performed for equivocal imaging findings while on treatment. Figure 3h shows immunohistochemical analysis demonstrating 24 CD3^+^ T cells per high-powered field present in the peritumoral space and within the tumor parenchyma. After completing the initial six doses of ELI-002 2P, patient 2 received subsequent treatment with an investigational checkpoint antibody off protocol, resulting in a radiographic partial response. In patient 18 (PDAC) treated on cohort 3 (2.5-mg Amph-CpG dose level), ctDNA returned to detectable levels after a 5-month period of MRD clearance. Subsequent imaging showed a lesion suspicious for recurrent disease, and biopsy was performed (Fig. 3i) showing 76 CD3^+^ T cells per high-powered field present throughout the tumor parenchyma. Patient 18 also received subsequent therapy including checkpoint inhibitor therapy, resulting in complete ctDNA clearance. Patient 16 underwent excisional biopsy showing T cell infiltration and continued study treatment for clinical benefit.

**Fig. 4:**
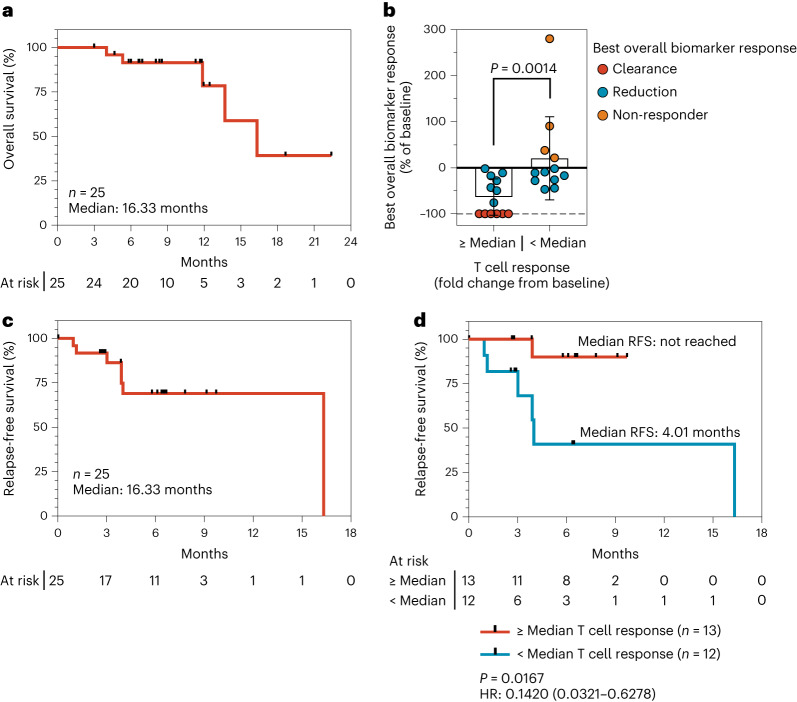
mKRAS T cell response correlates to tumor biomarker response and delayed tumor recurrence. **a**, OS from study start (date of first vaccine dose), *n* = 25; *n* indicates individual patients. **b**, Best overall tumor biomarker response for study participants reported as percentage of the baseline value, stratified by T cell response (maximum fold change from baseline; ≥ median versus < median), *n* = 25. *P* value was calculated by two-tailed Mann–Whitney test. **c**, RFS from study start (date of first vaccine dose), *n* = 25; n indicates individual patients. Values depicted are mean ± s.d. **d**, RFS from study start (date of first vaccine dose) stratified by T cell response (maximum fold change from baseline; ≥median versus <median), *n* = 25; *n* indicates individual patients. HR indicates hazard ratio with 95% CI. *P* values were calculated using two-tailed log-rank test.

**Extended Data Fig. 6:**
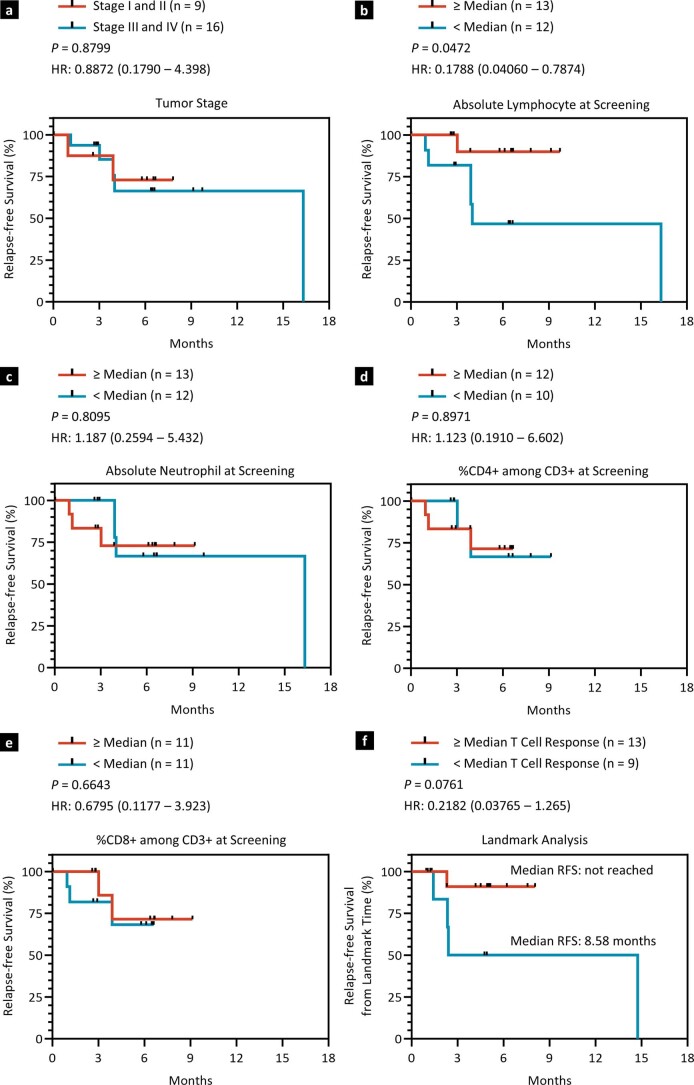
Baseline prognostic values and landmark RFS analysis. **a-e**, RFS from study start (date of first vaccine dose) stratified by **a**, tumor stage at baseline, *n* = 25, **b**, absolute lymphocyte count in peripheral blood at baseline, *n* = 25, **c**, absolute neutrophil count in peripheral blood at baseline, *n* = 25, **d**, %CD4+ among CD3+ lymphocytes at baseline among ICS evaluable patients, *n* = 22, **e**, %CD8+ among CD3+ lymphocytes at baseline among ICS evaluable patients, *n* = 22. **f**, RFS from landmark (completion of prime immunization) stratified by T cell response (Maximum fold change from baseline; ≥ Median versus < Median) among evaluable patients with RFS exceeding the landmark as of the data cut-off, *n* = 22. n indicates individual patients. HR indicates hazard ratio with 95% CI. P values calculated using two-tailed log-rank test.

**Extended Data Fig. 7:**
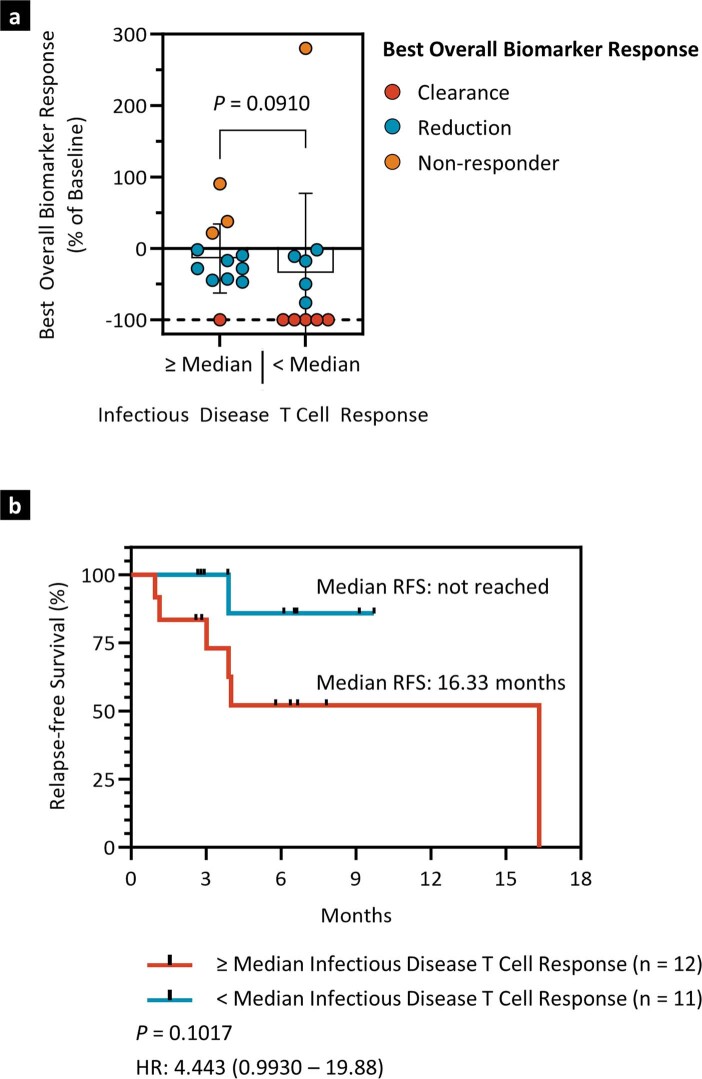
T cell response to infectious disease epitopes does not correlate to tumor biomarker response or delayed tumor recurrence. **a**, Best overall biomarker response for study participants reported as percentage of the baseline value, stratified by infectious disease epitope-specific T cell response (≥Median versus < Median), *n* = 23. Values depicted are mean ± standard deviation. P value calculated by two-tailed Mann-Whitney test. **b**, RFS from study start (date of first vaccine dose) stratified by infectious disease epitope-specific T cell response (≥Median versus < Median), *n* = 23. *n* indicates individual patients. HR indicates hazard ratio with 95% CI. P value calculated using two-tailed log-rank test.

At 8.5-months median follow-up of the cohort, the exploratory endpoints of RFS and overall survival (OS) were assessed. The median OS was 16.33 months (Fig. 4a). To assess the association of ELI-002 2P-induced expansion of mKRAS-specific immunity with tumor biomarker and RFS endpoints, T cell responses were classified as above median (fold change from baseline ≥12.75-fold; *n* = 13/25, 52%) or below median (<12.75-fold; *n* = 12/25, 48%). Above-median T cell response correlated with biomarker-assessed clinical anti-tumor activity (Fig. 4b), with a median change from baseline in tumor biomarker of −76.0%, whereas below-median T cell responders exhibited more frequent tumor biomarker progression and less frequent reduction or clearance (median change from baseline of −10.2%; *P* = 0.0014). Among above-median T cell responders, 100% (13/13) showed biomarker reduction; 46% (6/13) achieved biomarker clearance; and none was a biomarker non-responder. In contrast, no below-median T cell responders achieved clearance, with 66.7% (8/12) showing reduction. These findings were further correlated to RFS. The median RFS for the study was 16.33 months (Fig. 4c). By contrast, the median RFS was not reached compared to 4.01 months in above-median versus below-median T cell responders, respectively (hazard ratio (HR) = 0.14, 95% confidence interval (CI) 0.03–0.63, *P* = 0.0167; Fig. 4d). To assess a time-to-response bias^29^ RFS was analyzed in patients who were recurrence free (*n* = 22) upon completion of ELI-002 priming immunization. This analysis showed favorable outcomes for patients with above-median versus below-median 12.75-fold T cell response (HR = 0.22, 95% CI 0.038–1.27, *P* = 0.076), suggesting that time-to-response bias did not have a substantial effect on the association of the mechanism of action T cell biomarker to RFS (Extended Data Fig. 6). To assess whether above-median T cell response was confounded by factors potentially associated with favorable prognosis^30^, we examined tumor stage and baseline immunologic parameters, including percentages of peripheral blood CD4^+^ and CD8^+^ T cells, peripheral blood neutrophil and total lymphocyte levels; tumor stage, baseline neutrophils and CD4^+^ and CD8^+^ T cells did not correlate with RFS (Extended Data Fig. 6). Among baseline parameters, only absolute lymphocyte level was associated with RFS, suggesting that lymphocyte recovery from previous cytotoxic treatment may facilitate vaccine response. Further analysis of baseline responses to a panel of T cell epitopes from common infectious pathogens (cytomegalovirus (CMV), Epstein–Barr virus (EBV), influenza, severe acute respiratory syndrome coronavirus 2 (SARS-CoV-2) and tetanus) indicated that the level of these responses was not significantly associated with tumor biomarker reduction or RFS (Extended Data Fig. 7). Taken together, these results show the induction of mKRAS-specific T cell expansion by ELI-002 2P correlated with reductions and clearance of tumor biomarkers and RFS.

## Discussion

In this first-in-human phase 1 trial, we demonstrated that lymph-node-directed ELI-002 2P vaccination targeting KRAS driver oncogenes present in one-quarter of solid tumors is immunogenic in 84% of patients with MRD^+^ relapse after locoregional treatment. T cell responses required no selected HLA patient restrictions and correlated with reduction and clearance of tumor biomarkers and a significantly improved RFS in patients with above-median T cell responses (not reached versus 4.01 months). These results are promising as detectable ctDNA or persistently elevated CA19-9 after definitive PDAC therapy leads to rapid relapse and universal mortality^31–34^. Similarly unfavorable outcomes occur in resected MRD^+^ CRC^35–37^. However, recently, treatment with a personalized neoantigen vaccine administered in combination with checkpoint inhibitor and cytotoxic chemotherapy observed T cell responses that associated with prolonged RFS after adjuvant therapy in pancreatic cancer^38^. Therefore, the use of vaccination to induce tumor-specific T cell responses targeting diverse neoantigens may reduce the risk of solid tumor relapse. The results from AMPLIFY-201 further demonstrate the potential for ELI-002 2P monotherapy to elicit high-magnitude public neoantigen-specific T cell responses in a large fraction of treated patients despite prior chemotherapy, with favorable off-the-shelf availability.

Previous clinical trial use of soluble TLR9 agonist CpG-7909 oligonucleotide was hampered by DLTs resembling cytokine release syndrome^39^. Likewise, subcutaneous administration of oligonucleotide-containing drugs show similar toxicities^40^. Compared to unmodified CpG, preferential biodistribution of Amph-CpG in lymphatics has been associated with reduced adverse events resulting from systemic exposure in mice^15,20^. Consistent with this, ELI-002 2P was administered safely with escalating Amph-CpG-7909 subcutaneous doses over a 100-fold range (0.1 mg through 10 mg).

Despite the potent cellular immune responses induced, no T-cell-related side effects, such as cytokine release syndrome, were observed. Furthermore, no autoimmune-related safety events were observed, and several patients enrolled with mild autoimmune conditions (for example, pernicious anemia, hyperparathyroidism and Grave’s disease) experienced no change in their disease. As expected for subcutaneous administration, the primary toxicities of ELI-002 2P were low-grade local and systemic effects common with vaccine administration, including fatigue, injection site reaction and myalgia^40^. Therefore, the Amph platform could be used to improve the safety of vaccines or immunotherapies by reprogramming biodistribution to restrict drug exposure and resulting immune activation to the lymph nodes.

The RP2D of Amph-CpG-7909 was 10 mg (cohort 5), which was well tolerated, with consistent tumor biomarker reductions and induction of mKRAS-specific T cell responses. All patients at the highest dose level, the selected 10-mg Amph-CpG RP2D, exhibited both tumor biomarker reduction and induction of mKRAS-specific T cells. The dose level of Amph-Peptides 2P was selected for initial evaluation based on prior clinical experience with similar unmodified mKRAS peptide immunogens^41^. Further assessment of safety and activity for Amph-Peptide dose level is ongoing in the AMPLIFY-7P trial (NCT05726864), which is evaluating a broad-spectrum seven-valent formulation of ELI-002.

We designed a clinical/translational trial that used recent advances in molecular diagnostics to enroll patients with MRD, defined for this protocol as ctDNA positivity and/or rising serum tumor antigen levels. Although ctDNA assessment allowed for detection of biomarker clearance (0 MTM/ml), we did not report any clearances for serum tumor biomarker reductions, as CA19-9 or CEA returned to the normal range but remained detectable. The trial design also provided an opportunity to maximize the ratio of effector T cells to target tumor cells in patients with MRD, a strategy considered to be particularly important in immunotherapy-recalcitrant malignancies. Strategizing to treat at this relatively early stage may preempt tumor-acquired immunotherapy resistance mechanisms, including loss of HLA class I expression^22^.

ELI-002 2P mKRAS Amph-peptides were designed to contain both 9-mer and 10-mer HLA class I epitopes as well as longer class II epitopes. This design supported development of both CD4^+^ and CD8^+^ T cell responses in 59% of patients. Beyond the anti-tumor potential for CD8^+^ T cells, the role for CD4^+^ T cells includes support of CD8^+^ T cell activation and direct cytotoxic activity^42,43^. The induction of a balanced CD4^+^ and CD8^+^ T cell response by ELI-002 2P is likely to support the development of a multi-pronged anti-tumor defense.

We did not observe evidence that the association of ELI-002 2P-induced T cell response with delayed recurrence was confounded by known prognostic baseline characteristics, such as tumor stage, or baseline immunologic parameters and pre-existing levels of T cells responding to common pathogens, such as CMV, EBV, SARS-CoV-2, influenza or tetanus. However, RFS and baseline absolute lymphocyte count were associated, suggesting that complete hematologic recovery from prior cytotoxic therapy may be required for optimal vaccine response.

Although adoptive T cell approaches have shown promising clinical activity, addressable patient populations have so far been restricted to select KRAS mutant and patient HLA combinations. In contrast, an effective vaccination strategy provides the opportunity to simultaneously expand T cells specific to multiple KRAS mutants restricted to a variety of HLA class I and class II through endogenous processing and presentation of vaccine immunogens. Although further evaluation among a larger patient population is needed, the initial observations with ELI-002 2P suggest that vaccine-induced T cell reactivities and anti-tumor responses were not limited to patients with known mKRAS-peptide binding HLA class I. Specifically, class I HLA alleles (A*02:01, A*03:01, A*11:01, B*07:02 and C*08:02), previously reported to restrict CD8^+^ T cell responses to mKRAS G12D or G12R mutations, were not required for T cell response, and this result was not explained by induction of CD4^+^ rather than CD8^+^ responses. Therefore, Amph vaccination may facilitate development of broad responses through previously unknown HLA restrictions (class I or class II)^11,23,24^. Analysis of these responses may yield new mKRAS T cell epitopes important for expanding understanding of mKRAS-directed immunity.

Another interesting aspect of this study is the observation of cross-reactive T cells to non-immunizing mKRAS antigens. Whether this represents T cell cross-reactivity or development of antigen specificity independent of G12R and G12D cannot be determined at this time. However, cross-reactive mKRAS-specific T cell receptors (TCRs) have been recently reported with co-recognition of G12D and G12V^44^. Separately, crystal structures show that amino acid position 12 can act as a major histocompatibility complex (MHC) anchor without contact to cognate TCR CDR3, indicating a mechanism for cross-reactive responses dependent on shared epitopes among distinct mKRAS sequences^24,45^. In addition, antigen binding to MHC class II molecules can be highly promiscuous, leading to cross-reactivity to TCRs of different specificities^24,45–48^. The clinical importance of broad mKRAS-specific immunity has been highlighted recently through analyses of acquired resistance to G12C inhibition where emergence of secondary mutations at position 12 resulted in tumor progression^49^. Further evaluation of the observed responses will be necessary to define the critical mechanisms important for restricting T cell cross-reactivity. Regardless of underlying mechanism, the potential to elicit broad immune recognition of mKRAS through vaccination holds potential to increase antigenic coverage and help prevent antigen escape, leading to more durable benefit.

Previous reports suggest that mKRAS-targeting T cell responses are not subject to central or peripheral tolerance^25,26^; often, responses show fidelity to the specific primary tumor KRAS mutation. Moreover, mKRAS-specific T cells have been isolated from TILs and peripheral blood in patients with CRC and in patients with non-small cell lung cancer (NSCLC), showing that T cells can localize and infiltrate tumors^12,26–28^. Consistent with these findings, we observed that 14 of 21 immune responders exhibited baseline mKRAS-specific T cell responses. These were substantially expanded after ELI-002 2P vaccination, demonstrating the potential to stimulate expansion of highly functional pre-existing T cell response through Amph vaccination. In addition, 100% of the subset of immune responders (7/7) with no detectable pre-existing T cell responses also developed de novo responses after ELI-002 2P vaccination, associated with tumor biomarker increase (1/7), reduction (4/7) or clearance (2/7). The expansion of pre-existing and de novo T cell responses observed here demonstrates that ELI-002 2P effectively amplifies mKRAS-specific T cells to expand their number and promote their functional quality—attributes that have correlated with clinical benefit for prior TCR-T and TIL-based therapies targeting mKRAS-driven cancers.

Recently, individualized neoantigen cancer vaccines have shown promising efficacy for PDAC, NSCLC, CRC and melanoma^38,48,50,51^. The availability of ELI-002 as an ‘off-the-shelf’ product offers several further advantages, including streamlined standard manufacturing to facilitate on-demand availability while eliminating the need for tumor-informed production, which presents operational risks and limits use to post-surgical adjuvant stage. Of further interest is the hypothetical potential for ELI-002 to augment the development of T cells specific for personalized tumor neoantigens. Induction of these responses through personalized vaccination has demonstrated their potential for anti-tumor activity in PDAC. Antigen spreading has been observed in previous preclinical studies of lymph-node-targeted Amph vaccine therapy for solid tumors, suggesting a potential for future analysis to explore this effect in clinical study of ELI-002^19,52–54^.

Recent studies have shown that mKRAS-targeted kinase inhibitor efficacy is dependent on T cell activity to achieve full anti-tumor effect, and T cell vaccine combinations with checkpoint inhibition have demonstrated promise^50,55^. Alongside the T cell induction observed with ELI-002 2P, these findings motivate the future development of immunotherapy or targeted therapy combination regimens to promote synergy and further improve clinical outcomes. In support of the rationale for checkpoint combination, two patients who were observed to have tumor-infiltrating CD3^+^ T cells after ELI-002 2P administration subsequently cleared ctDNA after checkpoint inhibitor therapy. This is notable given that multiple previous studies of checkpoint inhibition in pancreatic cancer have observed a 0% response rate^5–8^, consistent with previous observation of poor T cell infiltration in these tumor types^13,56^.

We acknowledge several limitations of our study, including small sample size, limited follow-up duration to date, absence of a control arm and the use of exploratory assessments for initial evaluation of pharmacologic activity of ELI-002 2P. Most patients were White, potentially limiting assessment of race and ancestry in relation to immune response. No Black patients enrolled; however, among two Asian patients and two patients who did not self-report racial background, all four exhibited T cell response. Although these findings are promising, additional studies including larger patient numbers and greater patient diversity will be needed to assess the potential for general applicability of ELI-002. Tumor biomarker reductions from baseline occurred in similar proportions assessed using CEA/CA 9-9 (9/12, 75%) and assessed using ctDNA (12/13, 92%). However, 50% CEA/CA19-9 responses were less frequent in one of 12 (8.3%) compared to 50% ctDNA responses that were observed in seven of 13 (53.8%) patients. In adjuvant CRC, even transient ctDNA clearance has been associated with increased disease-free survival^37^. Larger future studies will be needed to separately analyze ctDNA and serum tumor biomarker responses, which may be biologically different, and to compare outcomes in those with sustained or transient reductions or at different response thresholds. Because no pre-treatment biopsy tissue was available for comparison, interpretation of the CD3^+^ T cell infiltration observed on the subset of six patients who underwent biopsies is limited. Finally, treatment was conducted with a constant Amph-Peptide-2P dose, preventing assessment of the impact of peptide dose level on study outcomes.

Overall, this study provides important proof of concept for the safety and immunogenicity of lymph-node-targeting Amph vaccines and yielded promising signals of clinical activity that correlate with the magnitude of ELI-002 2P-induced T cell response. These findings demonstrate the hypothesized mechanism of action for the lymph-node-targeting Amph platform and suggest potential for use whenever pharmacological T cell responses are required, spanning indications in infectious disease as well as oncology. Despite the limitations noted for this single-arm, signal-seeking phase 1 design, which precludes assessment of causality, the preliminary signals observed warrant a randomized phase 2 study. Development of ELI-002 is proceeding with a phase 1 and randomized phase 2 study (NCT05726864) of a seven-peptide formulation (ELI-002 7P: KRAS/NRAS G12D, R, V, S, A, C and G13D). This will offer additional opportunities to evaluate the activity of Amph vaccines in malignancies driven by a broad spectrum of KRAS mutations.

## Methods

### Study design, patients, treatment and oversight

A phase 1, multi-center, open-label, first-in-human trial of ELI-002 2P monotherapy was conducted in five ascending dose cohorts at seven centers in the United States between 4 October 2021 and 6 September 2023 (the clinical cutoff date for the results presented here). A fixed dose of Amph-Peptides 2P (G12D and G12R, 0.7 mg each) was administered with escalating doses of 0.1, 0.5, 2.5, 5.0 and 10.0 mg of Amph-CpG-7909 adjuvant. Eligible patients were 18 years of age or older, had mKRAS G12D-mutated or G12R-mutated pancreatic or colorectal cancers and were at high risk for relapse because of the presence of MRD (indicated by ctDNA positivity or elevated serum CA19-9 and/or CEA). Academic centers entered clinical data into Medidata RAVE 2018.2.4. Additional details are provided in the study protocol (Supplementary Data 1).

Two participating institutions, the University of Colorado School of Medicine and City of Hope, had the study approved by WIRB Copernicus (WCG IRB). Six other participating institutions had the study approved by their local IRBs: Memorial Sloan Kettering Cancer Center (MSKCC IRB), The University of Texas MD Anderson (University of Texas MD Anderson Office of Human Subject Protection), the University of Iowa (University of Iowa Human Subjects Office/IRB), Northwell Health (Feinstein Institutes for Medical Research, Northwell Health IRB), the University of California, Los Angeles (UCLA Office of the Human Research Protection Program) and Massachusetts General Hospital (Dana-Farber Cancer Institute Office for Human Research Studies). The study was approved by the US Food and Drug Administration (FDA) and registered on ClinicalTrials.gov (NCT04853017).

### Patients

We enrolled adult (≥18 years of age) patients with Eastern Cooperative Oncology Group (ECOG) performance status (PS) of 0 or 1 with pathologically confirmed mKRAS (G12D or G12R) PDAC or CRC who were MRD^+^ with either (1) absolute CA19-9 ≥ 90 U ml^−1^ or CEA ≥ 15 ng ml^−1^ or (2) successively rising values (≥1 week apart) in either CA19-9 or CEA not attributable to a non-cancer condition, such as pancreatitis, peritonitis, postoperative leak/fistula or biliary obstruction. Patients had recovered from prior surgery, chemotherapy or radiation without ongoing medical/surgical issues and were willing to use effective methods to avoid pregnancy and provided written informed consent. Baseline absolute neutrophil count ≥1.5 × 10^9^/L, platelets ≥100 × 10^9^/L, normal range liver function tests, serum creatinine <1.5 (or if serum creatinine was ≥1.5 mg dl^−1^, creatinine clearance calculated by the Cockcroft–Gault formula ≥60 ml min^−1^ was acceptable), albumin ≥2.5 g dl^−1^ and IL-6 < 500 pg ml^−1^ were required.

Patients with PDAC had high-risk tumor stages I or II or III or oligometastatic stage IV disease per current American Joint Committee on Cancer (AJCC) criteria with no evidence of disease on current imaging (equivocal radiographic findings, such as subcentimeter lesions or potential resolving soft tissue changes after surgery, were accepted), prior treatment with standard chemotherapy/chemoradiation administered in the neoadjuvant and/or adjuvant setting and complete tumor resection (R0 or R1 pathologic margins), with focal use of intraoperative irreversible electroporation permitted.

Patients with CRC had high-risk stage II (T4N0), stage III (T4N1-2/TanyN2) or stage IV oligometastatic disease per current AJCC staging criteria, prior cytotoxic chemotherapy administered in the neoadjuvant or adjuvant setting, or as total neoadjuvant therapy, and complete surgical resection (R0 or R1 pathologic margins), with focal use of intraoperative irreversible electroporation permitted.

We excluded patients who had received anti-tumor therapy within 4 weeks; who had a history of brain metastasis; who had other malignancies within the past 3 years (except for adequately treated carcinoma of the cervix, bladder or prostate or basal or squamous cell skin cancer); who were receiving immunosuppressive drugs; who had serious comorbid illnesses, including uncontrolled infection, class III or class IV (New York Heart Association) cardiac failure, myocardial infarction within 6 months, active seizure disorders, autoimmune diseases or interstitial lung disease if requiring systemic steroids; who had pulse oximetry less than 92% on room air; who had prior organ transplants; and who had HIV/AIDS, hepatitis B or hepatitis C (unless they had a sustained virologic response to direct-acting antiviral therapy), and those who were in the first 2 weeks of SARS-CoV-2. Women were excluded if pregnant or lactating. Patients with PDAC were excluded when tumors were of neuroendocrine subtype or when there was a germline BRCA 1/2 mutation. Patients with CRC were excluded when tumors were mismatch repair defective (MSI^+^).

Treatment was divided into a Prime Immunization series (six subcutaneous doses of ELI-002 2P over 8 weeks), a 3-month No Dosing Period (observation) and a Booster Immunization series (four weekly doses of ELI-002 2P). A Follow-up Period included up to 2 years after the first dose of ELI-002 2P to monitor safety and efficacy (Extended Data Fig. 1).

The study was sponsored and designed by Elicio Therapeutics in collaboration with the academic authors. The study and analyses were conducted in accordance with the general principles of the Declaration of Helsinki and Good Clinical Practice guidelines of the International Council for Harmonization. The trial protocol, amendments and supporting documents were approved by the local/central IRB for each study site and the US FDA and were registered on ClinicalTrials.gov (NCT04853017). All patients provided written informed consent.

A safety and monitoring committee was convened to review safety and determine dose escalation and cohort expansion decisions. Cohorts ranged from three to six patients with expansions allowed after the first three patients completed 28 d without DLT and when additional eligible patients had been identified.

All authors affirm that the trial was conducted in accordance with the study protocol and vouch for the accuracy and completeness of the data. A professional medical writer funded by the sponsor aided in preparation of the first draft of the manuscript in accordance with Good Publication Practice guidelines. All authors reviewed and revised the manuscript and made the decision to submit it for publication.

The initial protocol (version 1.0) was approved on 13 July 2020. Key protocol amendments are as follows. Amendment 2 (version 3.0) was approved on 23 February 2021 and included changes requested by the FDA. This was the initial protocol for study initiation. On 8 April 2022, Amendment 4 (version 5.0) was approved and added serum tumor biomarkers (that is, CEA and CA19-9) to the MRD eligibility along with ctDNA. Amendment 5 (version 6.0), approved on 2 August 2022, added language regarding pseudo-progression and continued ELI-002 dosing. Amendment 6 (version 7.0) was approved on 25 January 2023 and added language for public record search for OS.

### Endpoints and assessments

Primary endpoints of the study were safety (adverse events were graded per Common Terminology Criteria for Adverse Events, version 5.0), tolerability and determination of the RP2D. The secondary endpoint was tumor biomarker reduction and clearance defined through assessment of ctDNA and/or serum tumor antigens (CA19-9 or CEA), and exploratory endpoints included radiographic RFS and immunogenicity. RFS and tumor biomarker endpoints were correlated with T cell biomarkers and high-resolution HLA typing.

### Immunogenicity analysis

PBMCs for immunogenicity analysis were processed from leukapheresis (baseline and week 9) or whole blood collections (all other timepoints). Patient PBMCs were processed by Ficoll-Hypaque gradient protocol for leukapheresis samples or cell processing tubes (BD Biosciences) for whole blood samples. PBMCs were resuspended in CS10 freezing media (Cryostor), frozen in aliquots of 10–20 million cells per cryovial and stored in a temperature-monitored liquid nitrogen vapor phase freezer.

### Ex vivo FluoroSpot assay

A direct IFNγ/GrB FluoroSpot assay was performed on thawed PBMCs. Cryopreserved PBMCs were thawed in 10% human AB serum/RPMI media + Benzonase and rested overnight at 37 °C. Pre-coated human IFNγ/GrB FluoroSpot plates were washed with PBS and blocked with AIM-V media for at least 30 min (MabTech). Then, 2 × 10^5^ rested PBMCs were plated into each well and stimulated for 44 h as per the manufacturer’s instructions with seven individual mKRAS peptide pools and a WT peptide pool (pools consisted of KRAS 18-mer peptide and 9-mer and 10-mer overlapping peptides (OLPs) of each KRAS 18mer; Supplementary Table 1) at 2 µg/peptide/ml. No exogenous cytokines were added to the PBMCs during this assay. All samples were plated in triplicate. Dimethyl sulfoxide was used as the negative control (background wells), and anti-CD3 (MabTech) was used as the positive control. The plate was developed based on the manufacturer’s instructions. Plates were scanned and counted using the IRIS plate reader (MabTech) using FITC and Cy3 filters (Supplementary Fig. 2). Data are background subtracted, averaged per triplicate measurements and normalized to 1 × 10^6^ PBMCs. A responder in the FluoroSpot assay was defined as a patient having ≥2-fold increase from baseline at any post-vaccination timepoint and more than 50 SFCs/1 × 10^6^ PBMCs. Baseline mKRAS-specific T cell responses were defined as more than 50 SFCs/1 × 10^6^ PBMCs to any mKRAS antigen in the FluoroSpot assay. The above ex vivo IFNγ/GrB FluoroSpot assay was also used to test T cell responses to infectious disease antigens. Baseline PBMCs were stimulated with 2 µg/peptide/ml of the following antigen pools from JPT Peptide Technologies: PepMix SARS-CoV-2 (S-RBD OLP pool); PepMix Pan-CMV Select (pool of 99 peptides, defined HLA class I- and II-restricted T cell epitopes from selected proteins of CMV); PepMix HCMVA pp65 (pp65 OLP pool); CEFS Ultra SuperStim Pool MHC-II subset (positive control pool of 68 known peptide epitopes for a broad range of HLA subtypes and different infectious agents for T cell stimulation of populations with a diverse ethnic background); CEFT pool (positive control pool of 27 peptides selected from defined HLA class-I restricted T cell epitopes from CMV, EBV, influenza and tetanus); PepMix Pan-EBV Select (virus-specific pool of 135 peptides (defined HLA class I- and II-restricted T cell epitopes) from selected proteins of EBV); and CEFX Ultra SuperStim Pool (positive control pool of 176 known peptide epitopes for a broad range of HLA subtypes and different infectious agents for T cell stimulation of populations with a diverse ethnic background).

### Ex vivo intracellular cytokine staining

A direct ICS assay for IL2, IFNγ and TNFα was performed by flow cytometry. PBMCs were thawed and rested overnight. Then, 10^6^ PBMCs per well were plated and stimulated for 17 h at 37 °C with individual mKRAS peptide pools at 2 μg/ml/peptide (Supplementary Table 1). GolgiStop and GolgiPlug (BD Biosciences) were also added to each well. The next day, cells were surface stained with antibodies against CD4 (BV421, clone: SK3, BD Biosciences cat. no. 566907, 1:40), CD8 (BV786, clone: RPA-T8, BD Biosciences cat. no. 563823, 1:25), CD45RA (Alexa 700, clone: HI100, BioLegend cat. no. 304120, 1:25), CCR7 (PE-CF594, clone: 15053, BD Biosciences cat. no. 562381, 1:12.5), Aqua Live/Dead marker (Thermo Fisher Scientific cat. no. L34966, 1:200) and dump markers CD14 (PE-Cy5, clone: 61D3, Thermo Fisher Scientific cat. no. 15-0149-42, 1:200), CD16 (PE-Cy5, clone: 3G8, BioLegend cat. no. 302010, 1:200) and CD19 (PE-Cy5, clone: SJ25C1, BioLegend cat. no. 363042, 1:200). Cells were subsequently fixed with CytoFix/CytoPerm (BD Biosciences) and further stained with antibodies against CD3 (APC-H7, clone: SK7, BD Biosciences cat. no. 560176, 1:40), IFNγ (FITC, clone: Mab11, BioLegend cat. no. 506504, 1:200), TNFα (BV711, clone: B27, BioLegend cat. no. 502940, 1:50) and IL2 (BV650, clone: MQ1-17H12, BioLegend cat. no. 502940, 1:50). Cells fixed in 0.5% formaldehyde were acquired on a BD FACSymphony, and data were analyzed with BD Biosciences FlowJo version 10 software (gating progression and example plots in Supplementary Fig. 2). A responder in the ICS assay was defined as a patient having ≥2-fold increase from baseline at any post-vaccination timepoint and more than 0.1% cytokine positive.

### Tumor biomarker assessment and mutation identification

Comprehensive Genomic Profiling–Whole-Exome Sequencing was performed to determine whether the patient’s tumor harbored at least one of the two mKRAS alleles targeted by the ELI-002 2P (G12D or G12R). The Natera Signatera ctDNA test evaluated for the presence or absence of ctDNA. Whole-exome sequencing was performed on formalin-fixed, paraffin-embedded tumor samples with at least 20% tumor content confirmed by a pathologist under Central Lab Improvement Amendments (CLIA) and College of American Pathologists (CAP) guidelines. Genomic DNA was extracted from the patient’s normal (whole blood) and tumor tissue. Libraries of tumor and matched germline DNA were prepared, and exomic regions were captured. The assay was performed by target enrichments of the isolated DNA, followed by 440× coverage sequencing on a HiSeq 2500 or a NovaSeq 6000 (Illumina). Somatic single-nucleotide variants (SNVs) that were present in the tumor and absent in the germline were identified. A proprietary Natera algorithm selected a set of 16 SNVs to maximize the detectability of tumor DNA if present in plasma. Polymerase chain reaction (PCR) primers targeting the 16 personalized SNVs were designed and synthesized to be used to identify and track ctDNA in a patient’s plasma. Cell-free DNA was extracted from plasma and run in the multiplexed personalized PCR assay. Plasma samples with two or more SNVs detected above a predefined confidence threshold were deemed ctDNA positive, and ctDNA concentration was reported as mean tumor molecules per milliliter of plasma. In patients who did not have adequate tumor tissue, a plasma-based ctDNA assay for mKRAS variants was performed: Sysmex SafeSEQ RAS-RAF. Cell-free DNA was isolated from plasma, and a next-generation sequencing (NGS)-based assay that evaluated K/NRAS to detect SNVs was performed using a NextSeq 550 (Illumina). ctDNA concentration was reported as mutant molecules per variant and mutant allele frequency. Local testing was permitted if already available to confirm mKRAS status. Serum tumor biomarkers, CA19-9 and CEA were analyzed by study site local laboratories.

### HLA typing

High-resolution HLA class I A, B and C and HLA class II DRB1, DRB345, DQB1, DPB1, DQA1 and DPA1 typing was performed at LabCorp using whole blood. The method used targeted NGS. HLA allele interpretation was based on IMGT/HLA database version 3.35.0.

### Statistical analysis

Descriptive statistics were used to summarize demographic, medical history and safety data. Continuous variables were summarized using mean, s.d., median, minimum value and maximum value. Categorical variables were summarized using frequency counts and percentages. Tumor biomarker reduction/clearance was tested for association with categorical variables, such as high versus low T cell response, using the Mann–Whitney test. The Kaplan–Meier method was used to estimate the survival distributions. The log-rank test was used to compare the RFS between the high and low T cell responders. Graphs were created using GraphPad Prism version 9.4.

### Reporting summary

Further information on research design is available in the Nature Portfolio Reporting Summary linked to this article.

## Online content

Any methods, additional references, Nature Portfolio reporting summaries, source data, extended data, supplementary information, acknowledgements, peer review information; details of author contributions and competing interests; and statements of data and code availability are available at 10.1038/s41591-023-02760-3.

## Supplementary information

Supplementary Information Supplementary Figs. 1 and 2 and Supplementary Table 1.

Reporting Summary

Supplementary Data 1 Trial protocol

## Figures and Tables

**Extended Data Table 1:**
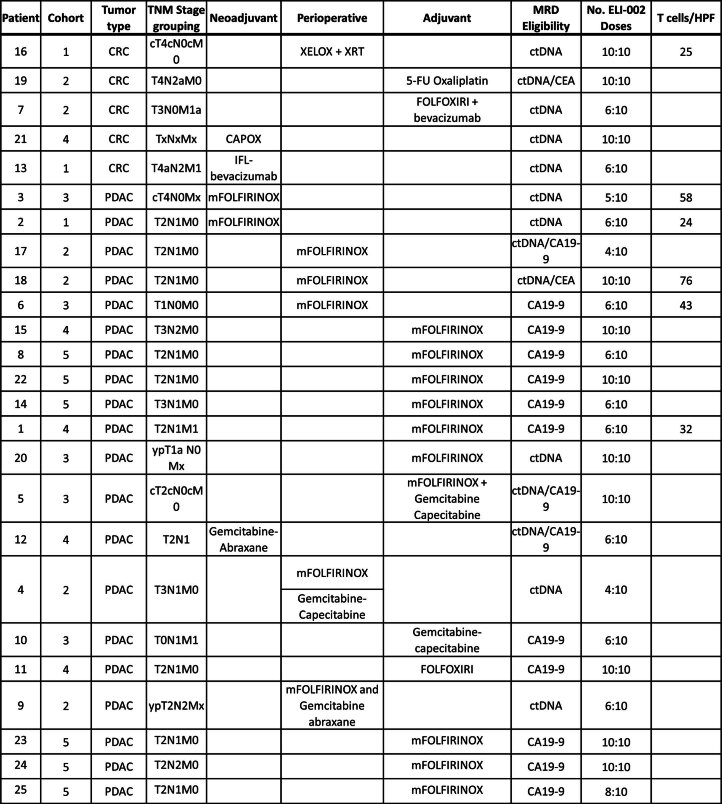
Individual patient characteristics

**Extended Data Table 2:**
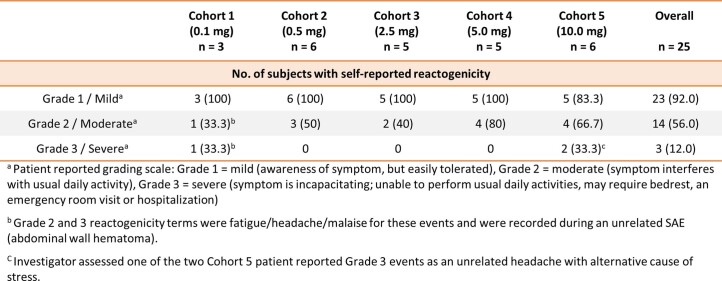
Patient-reported reactogenicity

**Extended Data Table 3:**
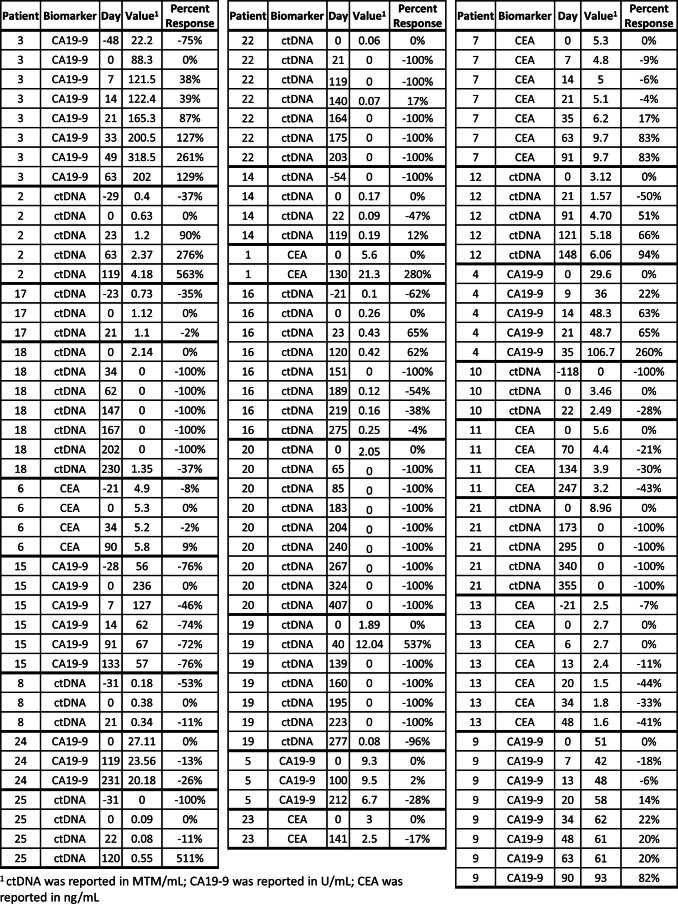
Patient biomarker values

Longitudinal biomarker values for patients collected during screening, treatment and follow-up. ctDNA was reported in MTM/ml; CA 19-9 was reported in U ml^−1^; and CEA was reported in ng ml^−1^.

## Data Availability

Requests must be made to datarequest@elicio.com, with responses provided within 30 d of request. To ensure that data sharing is consistent with the underlying study consent, de-identified patient data that can be shared will be done under data transfer agreements. Investigators and institutions who agree to the terms of the data transfer agreement, which will include, but will not be limited to, terms to address the use of these data for the purposes of a specific project and for research purposes only, to prohibit attempts to re-identify the data and to protect the confidentiality of the data, will be granted access to the data. Elicio Therapeutics will then facilitate the transfer of the requested de-identified data to the requestor using secure electronic data transmission. The data will then be available for up to 12 months.
