# Supplementary material for: Lymph-node-targeted, mKRAS-specific amphiphile vaccine in pancreatic and colorectal cancer: the phase 1 AMPLIFY-201 trial
Source: Nat Med. 2024 Jan 9;30(2):531–42. doi: 10.1038/s41591-023-02760-3 (PMC10878978; doi:10.1038/s41591-023-02760-3)
Supplement: Supplementary file 1 — Supplementary Figs. 1 and 2 and Supplementary Table 1. [file 41591_2023_2760_MOESM1_ESM.pdf]

nature medicine

Article

https://doi.org/10.1038/s41591-023-02760-3

Lymph-node-targeted, mKRAS-specific amphiphile vaccine in pancreatic and colorectal cancer: the phase 1 AMPLIFY-201 trial

In the format provided by the authors and unedited

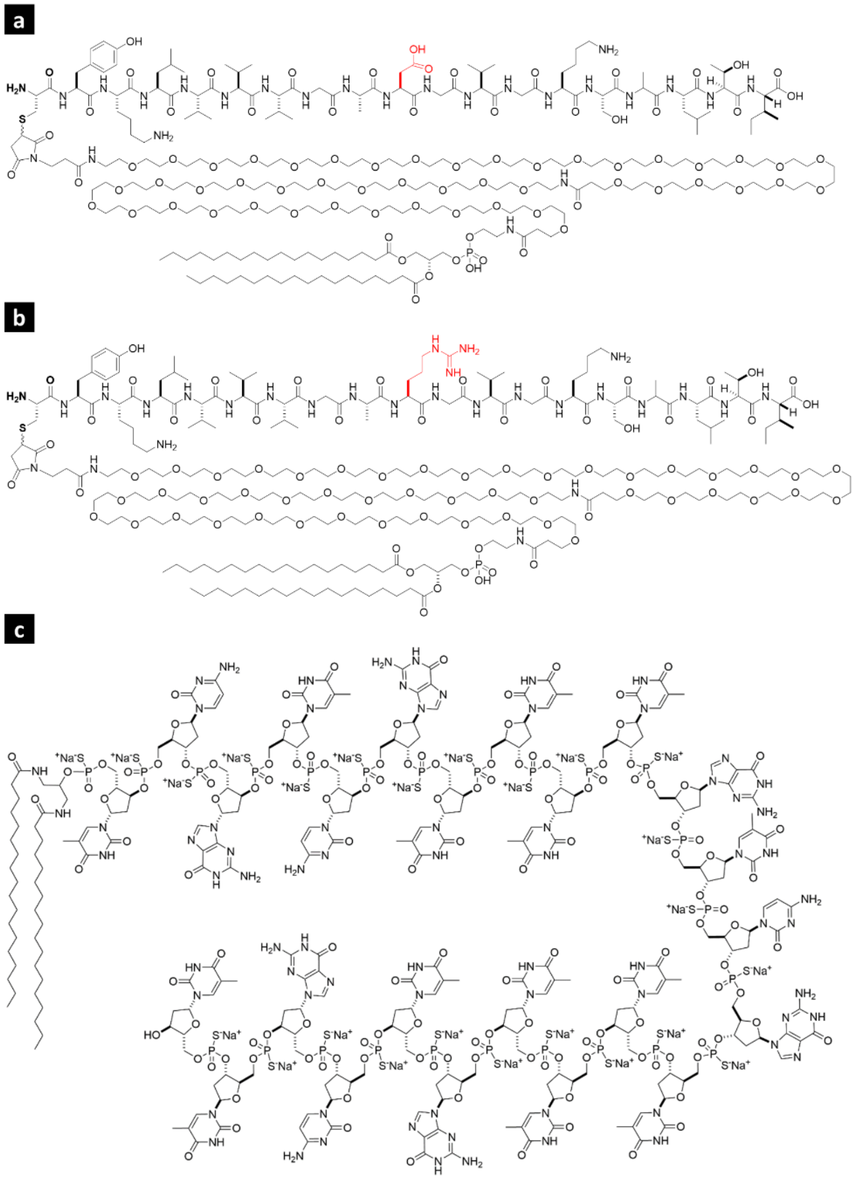

Supplementary Figure 1: Chemical structures for ELI-002 2P components.

Chemical structures for a, Amph-Peptide G12D, b, Amph-Peptide G12R, and c, Amph-CpG-7909. Amino acid side chains for G12D and G12R shown in red.

Peptide Name	Peptide Sequence	Peptide Length
G12D Long	YKLVVVGADGVGKSALTI	18
G12D Short 9-1	YKLVVVGAD	9
G12D Short 9-2	KLVVVGADG	9
G12D Short 9-3	LVVVGADGV	9
G12D Short 9-4	VVVGADGVG	9
G12D Short 9-5	VVGADGVGK	9
G12D Short 9-6	VGADGVGKS	9
G12D Short 9-7	GADGVGKSA	9
G12D Short 9-8	ADGVGKSAL	9
G12D Short 9-9	DGVGKSALT	9
G12D Short 10-1	YKLVVVGADG	10
G12D Short 10-2	KLVVVGADGV	10
G12D Short 10-3	LVVVGADGVG	10
G12D Short 10-4	VVVGADGVGK	10
G12D Short 10-5	VVGADGVGKS	10
G12D Short 10-6	VGADGVGKSA	10
G12D Short 10-7	GADGVGKSAL	10
G12D Short 10-8	ADGVGKSALT	10
G12D Short 10-9	DGVGKSALTI	10

Peptide Name	Peptide Sequence	Peptide Length
G12V Long	YKLVVVGAVGVGKSALTI	18
G12V Short 9-1	YKLVVVGAV	9
G12V Short 9-2	KLVVVGAVG	9
G12V Short 9-3	LVVVGAVGV	9
G12V Short 9-4	VVVGAVGVG	9
G12V Short 9-5	VVGAVGVGK	9
G12V Short 9-6	VGAVGVGKS	9
G12V Short 9-7	GAVGVGKSA	9
G12V Short 9-8	AVGVGKSAL	9
G12V Short 9-9	VGVGKSALT	9
G12V Short 10-1	YKLVVVGAVG	10
G12V Short 10-2	KLVVVGAVGV	10
G12V Short 10-3	LVVVGAVGVG	10
G12V Short 10-4	VVVGAVGVGK	10
G12V Short 10-5	VVGAVGVGKS	10
G12V Short 10-6	VGAVGVGKSA	10
G12V Short 10-7	GAVGVGKSAL	10
G12V Short 10-8	AVGVGKSALT	10
G12V Short 10-9	VGVGKSALTI	10

Peptide Name	Peptide Sequence	Peptide Length
G12R Long	YKLVVVGARGVGKSALTI	18
G12R Short 9-1	YKLVVVGAR	9
G12R Short 9-2	KLVVVGARG	9
G12R Short 9-3	LVVVGARGV	9
G12R Short 9-4	VVVGARGVG	9
G12R Short 9-5	VVGARGVGK	9
G12R Short 9-6	VGARGVGKS	9
G12R Short 9-7	GARGVGKSA	9
G12R Short 9-8	ARGVGKSAL	9
G12R Short 9-9	RGVGKSALT	9
G12R Short 10-1	YKLVVVGARG	10
G12R Short 10-2	KLVVVGARGV	10
G12R Short 10-3	LVVVGARGVG	10
G12R Short 10-4	VVVGARGVGK	10
G12R Short 10-5	VVGARGVGKS	10
G12R Short 10-6	VGARGVGKSA	10
G12R Short 10-7	GARGVGKSAL	10
G12R Short 10-8	ARGVGKSALT	10
G12R Short 10-9	RGVGKSALTI	10

Peptide Name	Peptide Sequence	Peptide Length
G12A Long	YKLVVVGAAGVGKSALTI	18
G12A Short 9-1	YKLVVVGAA	9
G12A Short 9-2	KLVVVGAAG	9
G12A Short 9-3	LVVVGAAGV	9
G12A Short 9-4	VVVGAAGVG	9
G12A Short 9-5	VVGAAGVGK	9
G12A Short 9-6	VGAAGVGKS	9
G12A Short 9-7	GAAGVGKSA	9
G12A Short 9-8	AAGVGKSAL	9
G12A Short 9-9	AGVGKSALT	9
G12A Short 10-1	YKLVVVGAAG	10
G12A Short 10-2	KLVVVGAAGV	10
G12A Short 10-3	LVVVGAAGVG	10
G12A Short 10-4	VVVGAAGVGK	10
G12A Short 10-5	VVGAAGVGKS	10
G12A Short 10-6	VGAAGVGKSA	10
G12A Short 10-7	GAAGVGKSAL	10
G12A Short 10-8	AAGVGKSALT	10
G12A Short 10-9	AGVGKSALTI	10

Peptide Name	Peptide Sequence	Peptide Length
G12S Long	YKLVVVGASGVGKSALTI	18
G12S Short 9-1	YKLVVVGAS	9
G12S Short 9-2	KLVVVGASG	9
G12S Short 9-3	LVVVGASGV	9
G12S Short 9-4	VVVGASGVG	9
G12S Short 9-5	VVGASGVGK	9
G12S Short 9-6	VGASGVGKS	9
G12S Short 9-7	GASGVGKSA	9
G12S Short 9-8	ASGVGKSAL	9
G12S Short 9-9	SGVGKSALT	9
G12S Short 10-1	YKLVVVGASG	10
G12S Short 10-2	KLVVVGASGV	10
G12S Short 10-3	LVVVGASGVG	10
G12S Short 10-4	VVVGASGVGK	10
G12S Short 10-5	VVGASGVGKS	10
G12S Short 10-6	VGASGVGKSA	10
G12S Short 10-7	GASGVGKSAL	10
G12S Short 10-8	ASGVGKSALT	10
G12S Short 10-9	SGVGKSALTI	10

Peptide Name	Peptide Sequence	Peptide Length
G12C Long	YKLVVVGACGVGKSALTI	18
G12C Short 9-1	YKLVVVGAC	9
G12C Short 9-2	KLVVVGACG	9
G12C Short 9-3	LVVVGACGV	9
G12C Short 9-4	VVVGACGVG	9
G12C Short 9-5	VVGACGVGK	9
G12C Short 9-6	VGACGVGKS	9
G12C Short 9-7	GACGVGKSA	9
G12C Short 9-8	ACGVGKSAL	9
G12C Short 9-9	CGVGKSALT	9
G12C Short 10-1	YKLVVVGACG	10
G12C Short 10-2	KLVVVGACGV	10
G12C Short 10-3	LVVVGACGVG	10
G12C Short 10-4	VVVGACGVGK	10
G12C Short 10-5	VVGACGVGKS	10
G12C Short 10-6	VGACGVGKSA	10
G12C Short 10-7	GACGVGKSAL	10
G12C Short 10-8	ACGVGKSALT	10
G12C Short 10-9	CGVGKSALTI	10

Peptide Name	Peptide Sequence	Peptide Length
G13D Long	YKLVVVGAGDVGKSALTI	18
G13D Short 9-1	KLVVVGAGD	9
G13D Short 9-2	LVVVGAGDV	9
G13D Short 9-3	VVVGAGDVG	9
G13D Short 9-4	VVGAGDVGK	9
G13D Short 9-5	VGAGDVGKS	9
G13D Short 9-6	GAGDVGKSA	9
G13D Short 9-7	AGDVGKSAL	9
G13D Short 9-8	GDVGKSALT	9
G13D Short 9-9	DVGKSALTI	9
G13D Short 10-1	YKLVVVGAGD	10
G13D Short 10-2	KLVVVGAGDV	10
G13D Short 10-3	LVVVGAGDVG	10
G13D Short 10-4	VVVGAGDVGK	10
G13D Short 10-5	VVGAGDVGKS	10
G13D Short 10-6	VGAGDVGKSA	10
G13D Short 10-7	GAGDVGKSAL	10
G13D Short 10-8	AGDVGKSALT	10
G13D Short 10-9	GDVGKSALTI	10

Peptide Name	Peptide Sequence	Peptide Length
WT Long	YKLVVVGAGGVGKSALTI	18
WT Short 9-1	YKLVVVGAG	9
WT Short 9-2	KLVVVGAGG	9
WT Short 9-3	LVVVGAGGV	9
WT Short 9-4	VVVGAGGVG	9
WT Short 9-5	VVGAGGVGK	9
WT Short 9-6	VGAGGVGKS	9
WT Short 9-7	GAGGVGKSA	9
WT Short 9-8	AGGVGKSAL	9
WT Short 9-9	GGVGKSALT	9
WT Short 9-10	GVGKSALTI	9
WT Short 10-1	YKLVVVGAGG	10
WT Short 10-2	KLVVVGAGGV	10
WT Short 10-3	LVVVGAGGVG	10
WT Short 10-4	VVVGAGGVGK	10
WT Short 10-5	VVGAGGVGKS	10
WT Short 10-6	VGAGGVGKSA	10
WT Short 10-7	GAGGVGKSAL	10
WT Short 10-8	AGGVGKSALT	10
WT Short 10-9	GGVGKSALTI	10

Supplementary Table 1: Stimulation peptides used for immunogenicity analysis.

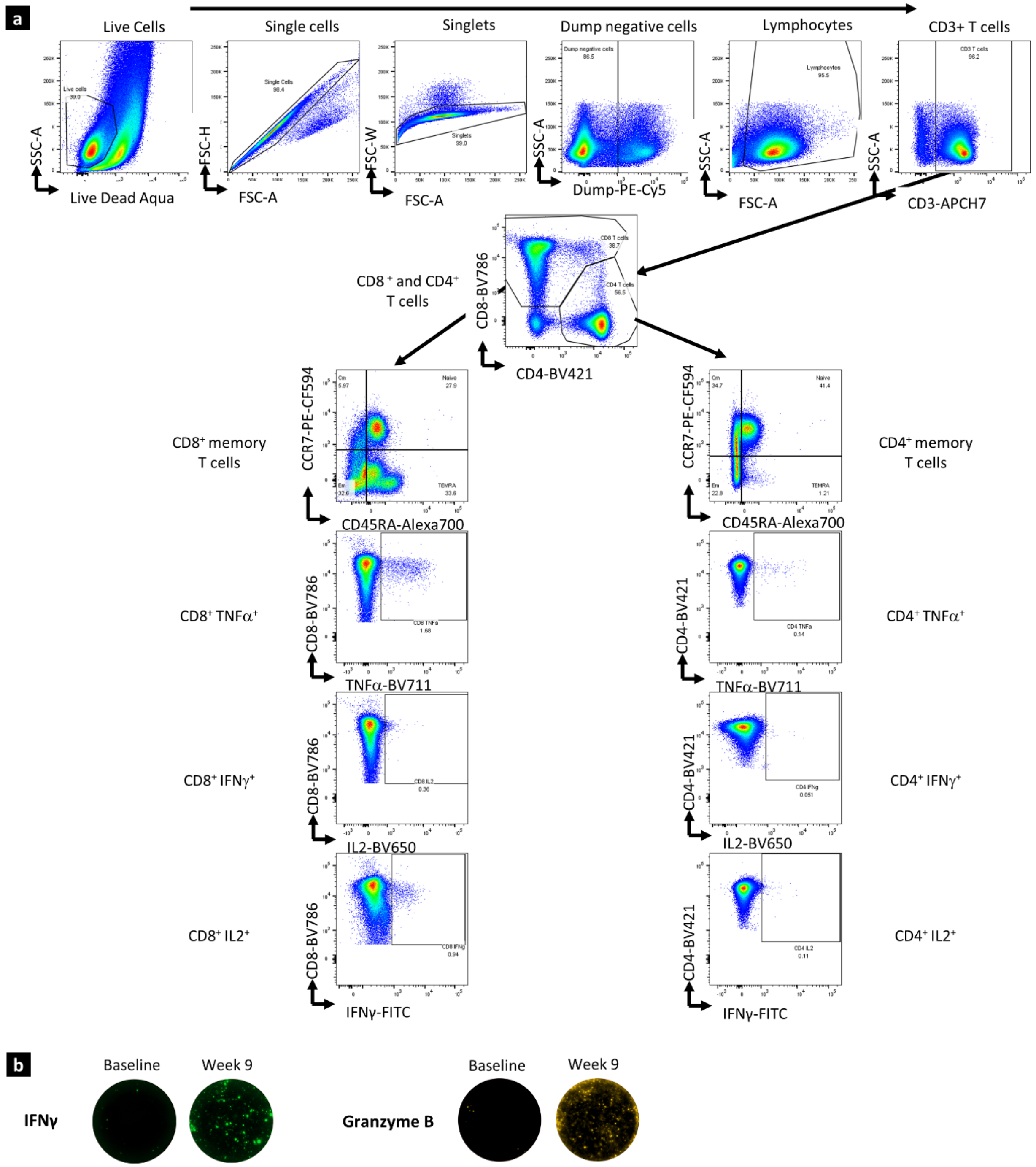

Supplementary Figure 2: ICS and memory phenotype gating strategy, example flow cytometry scatter plots, and example Fluorospot well images.

a, Gating strategy and example scatter plots for ICS assay and T cell memory phenotype assay for Patient 11. **b,** Example well images for baseline and week 9 PBMC IFNγ and Granzyme B Fluorospot assay.
